# The absence of *Trim28* in nephron progenitors results in impaired kidney development and function

**DOI:** 10.1242/dev.205188

**Published:** 2026-08-13

**Authors:** Balazs Palhazi, Alireza Paikari, Greta Clarén, Silke Appenzeller, Fabian Imdahl, Christoph Daniel, Matthias Becker, Manfred Gessler

**Affiliations:** ^1^ Department of Developmental Biochemistry, Theodor Boveri-Institute, University of Würzburg, D-97074 Würzburg, Germany; ^2^ Comprehensive Cancer Center Mainfranken, University Hospital of Würzburg, D-97080 Würzburg, Germany; ^3^ Helmholtz Institute for RNA-based Infection Research (HIRI), Single-Cell Center, D-97076 Würzburg, Germany; ^4^ Department of Nephropathology, Friedrich-Alexander Universität (FAU) Erlangen-Nürnberg, D-91054 Erlangen, Germany

**Keywords:** *Trim28*, Kidney development, Kidney tubule function, Transposable elements, Single-cell sequencing, Mouse

## Abstract

The *TRIM28* gene encodes a transcriptional regulator involved in a variety of molecular processes. Inactivation of *TRIM28* has been linked to the epithelial subtype of Wilms tumor, the most common pediatric renal malignancy. Therefore, TRIM28 may impact early kidney morphogenesis. To investigate its role in nephrogenesis, we analyzed *Trim28* conditional knockout (*Trim28*^Δ/Δ^) mice. We found that deleting *Trim28* in nephron progenitors resulted in early postnatal lethality and reduced kidney weight. While all renal structures were present, the proximal tubules (PTs) were disorganized. Transcriptome analyses revealed cell type-specific deregulation of retroelement expression following *Trim28* deletion. Further analysis of scRNA-seq data revealed a decreased frequency of nephron progenitor cells (NPCs), an increase in PT cells and a clear shift towards inflammatory gene expression patterns. While there was no evidence of tumor formation in *Trim28*^Δ/Δ^ kidneys, the differentially expressed genes in *Trim28*^Δ/Δ^ progenitors reflected reduced translation, similar to patterns observed in *TRIM28* mutant tumor-derived NPCs. Thus, our data demonstrate that TRIM28 primarily influences the maintenance and differentiation of NPCs and PT cells, as well as their subsequent function.

## Teaser

**Summary:** TRIM28 is a crucial factor for kidney development, and its knockout has specific effects on different renal cell types.

## INTRODUCTION

TRIM28 (also known as KAP1 or TIF1β) acts as a transcriptional co-repressor by interacting with DNA-binding zinc-finger proteins and chromatin modifying enzymes. An important function appears to be the repression of transposable elements (TEs) such as endogenous retroviruses (ERVs) (Trono, 2015). In this context, TRIM28 has been shown to bind to DNA indirectly via sequence-specific Krüppel-associated box zinc-finger proteins (KRAB-ZFPs). TRIM28 recruits a number of repressive chromatin factors such as members of the NuRD complex and the heterochromatin-associated protein HP1 (Sripathy et al., 2006). TRIM28-mediated silencing of retrotransposon-based enhancers appears to be essential for maintaining transcriptional repression of developmental gene expression programs in embryonic stem cells (ESCs) (Rowe et al., 2013). On the other hand, recent work describes a function of TRIM28 in oncogenic progression via RNA Pol II recruitment and pause release at hypoacetylated H4 tails (Bacon et al., 2020). TRIM28 has also been proposed to act both as a ubiquitin E3 ligase and as a SUMO ligase, with a range of substrates related to gene expression/cell cycle control and genomic integrity (Ivanov et al., 2007). *Trim28* plays a pivotal role in the early post-implantation development of mouse embryos, particularly during the egg-cylinder stage and the onset of gastrulation. Embryos lacking *Trim28* exhibit developmental arrest before mesoderm formation, accompanied by defects in the visceral endoderm. Its association with KRAB-ZFP-mediated gene silencing further suggests a broader role in regulating cell differentiation (Cammas et al., 2000).

A specific role for TRIM28 in the kidney has so far not been reported. However, inactivating *TRIM28* mutations have been associated with the formation of epithelial type nephroblastoma (Armstrong et al., 2018; Halliday et al., 2018). Nephroblastoma, also known as Wilms tumor (WT), is the most common kidney cancer in young children, affecting 1 in 10,000 children worldwide (Hohenstein et al., 2015; Spreafico et al., 2021). WT is thought to emerge through impaired renal development, and it is characterized by a wide range of histological patterns and a variety of genetic alterations. *TRIM28* mutations are genetic drivers in ∼5% of WTs and represent the second most common predisposing genetic event, following heterozygous inactivation (Wegert et al., 2025). This inactivation is particularly seen in epithelial-type WTs, where mutant *TRIM28* has emerged as a key predisposing gene. In a recent study of 126 epithelial-type WTs, 44.4% showed loss of TRIM28 expression by immunohistochemistry and 46% of these already carried predisposing mutations (Wegert et al., 2024). In the largest cohort of bilateral and familial WTs to date (129 patients), *TRIM28* was confirmed as the second most frequent predisposition gene, with affected tumors showing biallelic inactivation via 19q LOH as essentially the sole oncogenic alteration and a uniform epithelial histology (Wegert et al., 2025). Although WT due to germline *TRIM28* variants has been predominantly associated with maternal transmission, Whitworth et al. recently documented the first case arising from paternal inheritance, demonstrating that paternal carriers should also be included in genetic counseling (Whitworth et al., 2024).

In this study, we aimed to elucidate the role of *Trim28* in kidney development, to investigate the cell type-specific effects of its targeted loss, and to explore how this may contribute to renal dysfunction and/or tumor formation.

## RESULTS

### *Trim28* inactivation in NPCs disrupts nephron development

**Fig. 1.**
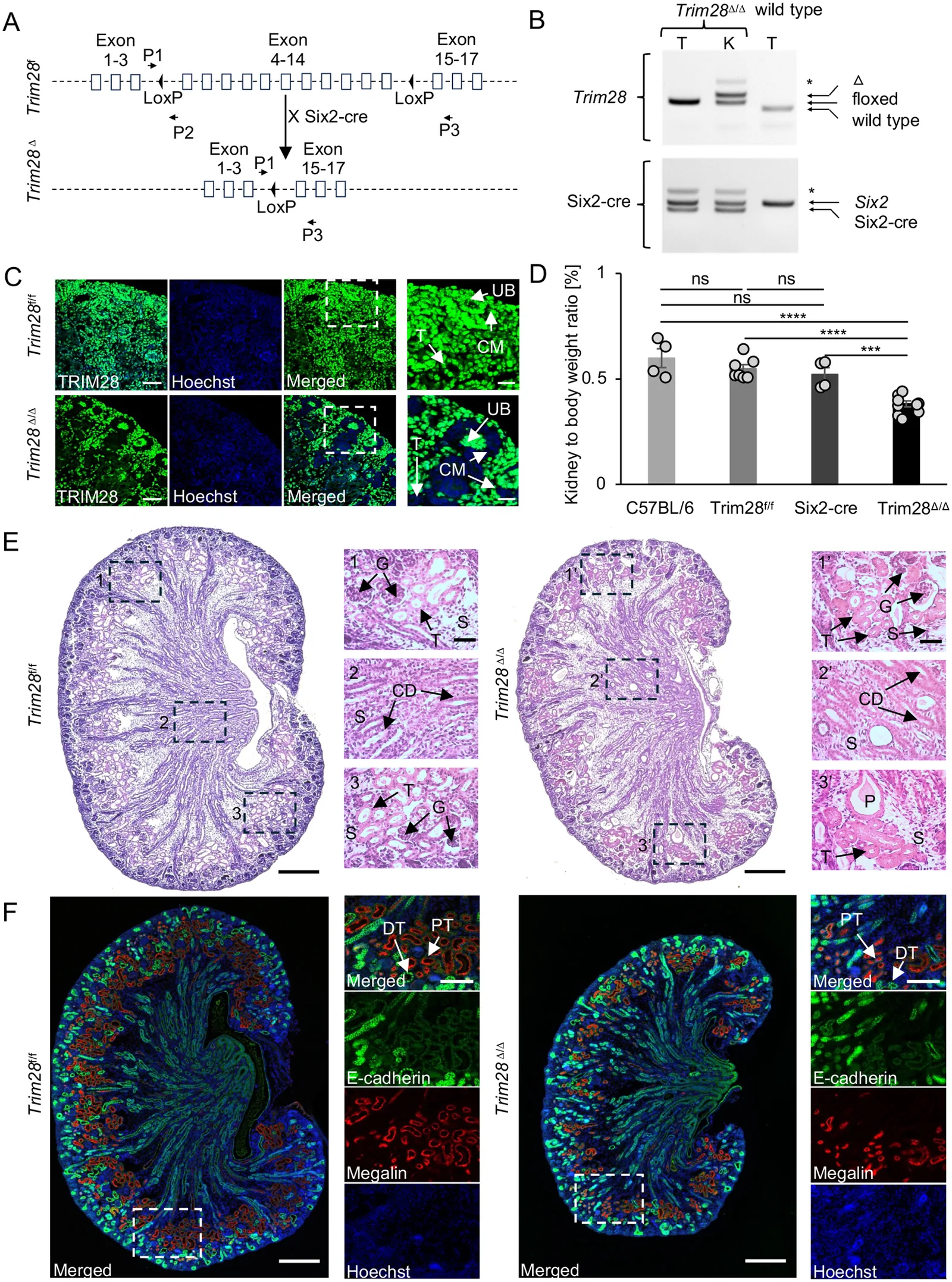
**Conditional deletion of *Trim28* and its renal phenotype.** (A) Schematic representation of the *Trim28^f^* allele and the *Trim28*^Δ^ allele following Cre-mediated recombination. P1-P3 indicate the location of primers used for genotyping of *Trim28* mutant mice. (B) PCR analysis of *Trim28*^f/f^/Six2-cre mouse tail (T) and kidney (K) samples at postnatal day 0 (P0), along with a wild-type control tail sample. The wild-type, floxed and recombined alleles (Δ) can easily be distinguished. Similarly, Six2-cre genotyping generated distinct wild-type and transgene products. Additional heteroduplex PCR products are indicated by an asterisk. (C) Immunofluorescent TRIM28 antibody staining of *Trim2*8^f/f^ and *Trim28*^Δ/Δ^ P0 mouse kidneys. Nuclei are counterstained with Hoechst 33342. Dashed boxes indicate areas of segment magnification shown on the right. Scale bars: 25 μm (left); 10 μm (magnification, right). (D) Comparison of the kidney-to-body weight ratio in wild-type C57BL/6, *Trim28*^f/f^, Six2-cre and *Trim28*^Δ/Δ^ mice. *Trim28*^Δ/Δ^ mice exhibit a significantly lower kidney-to-body weight ratio compared to controls. ****P<0.0001; ***P<0.001 (two-sided Welch's *t*-test). Data are mean±s.e.m. C57BL/6 (*n*=4), *Trim28*^f/f^ (*n*=7), Six2-Cre (*n*=4) and *Trim28*^Δ/Δ^ (*n*=9) mice were used. (E) H&E staining of P0 mouse kidneys reveals structural differences, including altered glomeruli, obstructed or thickened tubules, and tubules filled with protein cylinders. Arrows indicate various kidney structures and abnormalities. (F) Immunofluorescence staining of P0 mouse kidneys for E-cadherin (distal tubules) and megalin (proximal tubules) indicated by arrows. CD, collecting duct; CM, cap mesenchyme; DT, distal tubule; G, glomeruli; P, protein deposit; PT, proximal tubule; S, stroma; T, tubule; UB, ureteric bud. In E and F, dashed boxes indicate areas of segment magnification shown on the right. Scale bars: 250 μm (left); 100 μm (magnification, right). For immunofluorescence and H&E staining, representative examples from *n*=3 biological replicates are shown.

Since a complete *Trim28* knockout results in early embryonic lethality, conditional genetic approaches are essential to study its function during development (Cammas et al., 2000). We used a conditional mouse line, *Trim28*^tm1.1Ipc^ (*Trim28*^f^), that carries loxP recombination sites in introns 3 and 14 of the murine *Trim28* gene (Cammas et al., 2000). Cre-mediated deletion of the floxed segment results in a shortened *Trim28* gene consisting of exons 1-3 and 14-17 (Fig. 1A). This encodes a truncated protein (198 versus 835 amino acids) that lacks most of its functional domains and the resulting translational frameshift will induce nonsense-mediated mRNA decay. To investigate the effect of *Trim28* loss during mouse kidney development, we interbred *Trim28*^f^ mice with *Six2* promoter-driven EGFP-cre transgenic mice [Six2-cre; Tg(*Six2*-EGFP/cre)1Amc/J] (Kobayashi et al., 2008). This leads to recombination of *Trim28* in all nephron progenitor cells (NPCs) of double mutant offspring (Fig. 1B).

The heterozygotes (*Trim28*^*wt/Δ*^) exhibited neither early lethality nor any overt abnormalities. Moreover, their kidney-to-body weight ratio did not differ significantly from that of control animals (Fig. S9). In contrast, homozygous inactivation of *Trim28* (*Trim28*^Δ/Δ^) in mouse kidneys resulted in early postnatal lethality. The mice died within hours of being born. Their overall condition did not point to a possible cause of death, as they all displayed visible milk spots, indicating that they were well-fed. In addition to the kidneys, other organs could potentially be affected in *Trim28*^Δ/Δ^ animals. *Six2* expression is not limited to the developing kidney, but is also detected in multiple embryonic tissues, including the craniofacial mesenchyme, branchial arches, foregut and midgut regions, genital eminence, somites and limb mesenchyme (Oliver et al., 1995; Kobayashi et al., 2008). As *Trim28* is expressed at a significant level during embryogenesis and is essential for this process, it is plausible that *Six2-cre* and *Trim28* are co-expressed in some of these cell populations (Messerschmidt et al., 2012). However, no further behavioral or developmental abnormalities were observed in *Trim28*^Δ/Δ^ newborns compared to their *Trim28*^f/f^ control littermates. Consequently, we hypothesize that any potential effects in these tissues are likely to be subtle and unlikely to compromise viability. Based on this, we conclude that kidney failure is the most likely cause of death in *Trim28*^Δ/Δ^ animals. Quantitative real-time PCR (qRT-PCR) revealed a 50% global reduction of *Trim28* transcript levels in embryonic day (E) 18.5 conditional knockout kidneys, consistent with Six2-cre being expressed in the nephrogenic, but not the stromal, cell lineage (Fig. S1). Immunofluorescence staining of tissue sections showed that TRIM28 was expressed in mesenchymal precursors on the outer rim of newborn [postnatal day (P) 0] mouse kidneys, whereas NPCs of the cap mesenchyme that express *Six2* and all subsequent tubular structures were negative for TRIM28 as expected. The ureteric bud and stromal cells remained positive, as they originate from progenitor pools that lack *Six2* expression (Fig. 1C). These data showed that *Trim28* is lost from all early and fully developed nephron structures that are derived from *Six2*^+^ progenitor cells.

Comparison of newborn kidneys showed that knockout kidneys were smaller in size than those of control littermates. Kidneys from *Trim28*^Δ/Δ^ newborns reached only 70% of the kidney/body weight ratio seen in wild-type C57/BL6 mice (*P*<0.0001, two-sided Welch's *t*-test) and this reduction was similar when Six2-cre and *Trim28*^f/f^ littermates were used as controls. We saw small and non-significant changes comparing C57/BL6, Six2-cre and *Trim28*^f/f^ newborns, which may be due to minor differences in genetic background or statistical variations. A smaller, but significant impairment of kidney growth has been reported previously for the Six2-cre allele on a CD-1 background (Perl et al., 2024), but this was not seen in a recent study by Xue et al. (2025), who used the same C57/BL6 background as was used in our study. Thus, it appears that deletion of *Trim28* in NPCs leads to a specific reduction in kidney size (Fig. 1D).

Histological analysis revealed disorganized tubular structures in P0 knockout kidneys. Some of the nephrons showed obstructed or strongly reduced tubular lumens in the cortical region (Fig. 1E). Additionally, we observed dilated tubular structures in the medullary region of the kidneys, some apparently filled with protein deposition. Double staining for megalin (proximal tubules; PTs) and E-cadherin (distal tubules; DTs) revealed that the PTs, in particular, were structurally compromised, with reduced lumens, potentially as a result of epithelial thickening (Fig. 1F).

These data suggest that TRIM28 inactivation impairs nephrogenesis and the correct maturation of nephron structures, with a focus on the PT segment.

### Proximal tubule markers are deregulated in the postnatal kidney

**Fig. 2.**
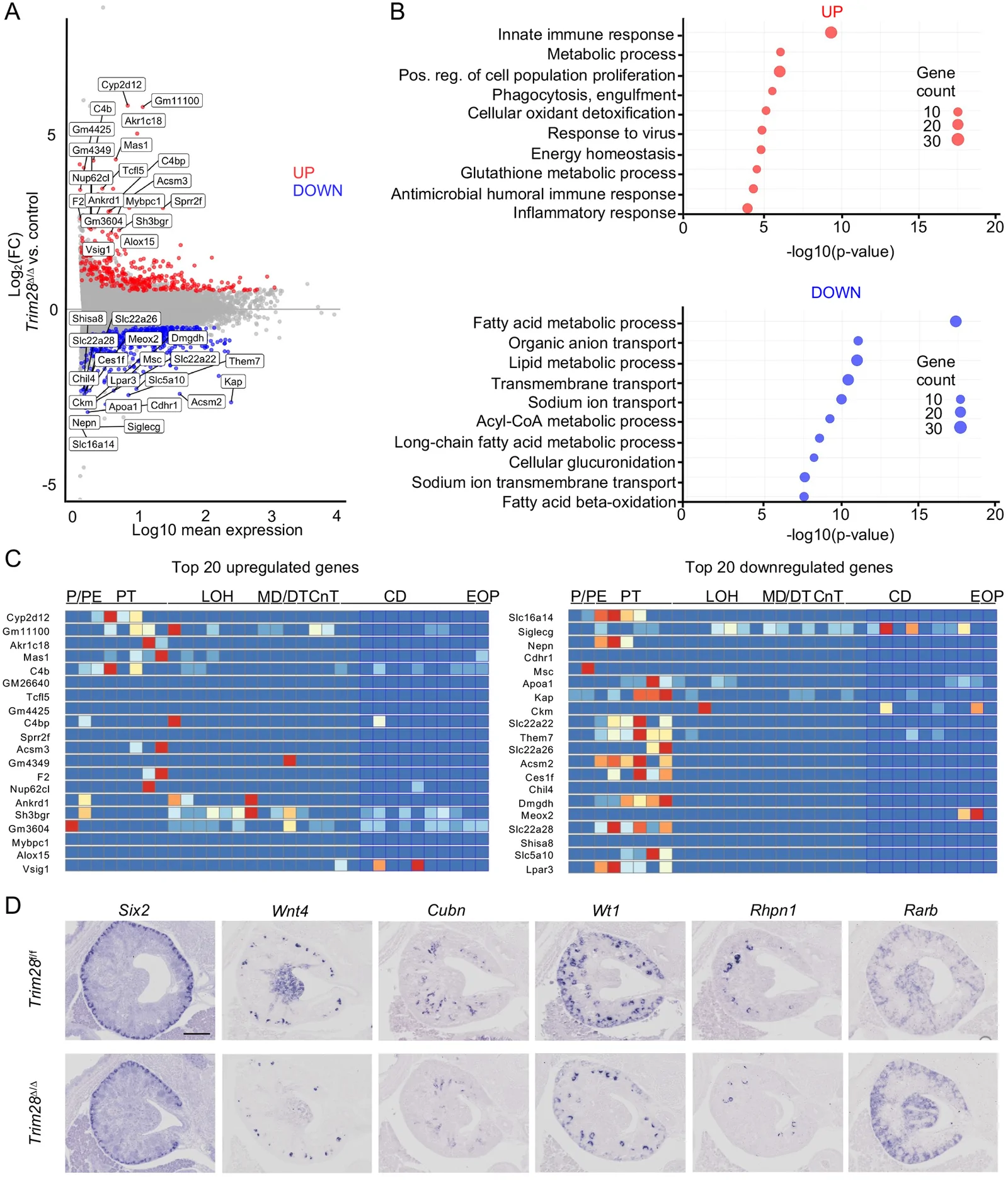
**Differential gene expression in *Trim28*^Δ/Δ^ kidneys.** (A) MA plot analysis of differentially expressed genes in bulk RNA-seq of P0 mouse kidney samples [*n*=3 per condition; *Trim28*^Δ/Δ^ versus control (*Trim28*^wt/f^ or *Trim28*^f/f^)]. The plot was generated in R v4.4.2 using standard functions from ggplot2 and ggrepel. Expression status was assigned based on the q-value and log_2_ fold change (log_2_FC). Genes with a q-value<0.05 and a log_2_(FC)>0.5 are highlighted in red (UP); genes with a q-value<0.05 and a log_2_(FC)<−0.5 are highlighted in blue (DOWN). The top 20 upregulated and downregulated genes are highlighted. (B) Top 10 GO terms of significantly upregulated and downregulated genes in P0 mouse kidneys according to the DAVID functional annotation tool (Huang et al., 2009; Sherman et al., 2022). (C) Heatmap analysis of the top 20 up- and downregulated genes mapped to nephron segments, based on single cell expression data from the Kidney Cell Explorer (https://cello.shinyapps.io/kidneycellexplorer/). P, podocytes; PE, parietal epithelium; PT, proximal tubule; LOH, loop of Henle; MD, macula densa; DT, distal tubule; CnT, connecting tubule; CD, collecting duct; EOP, deep medullary epithelium of pelvis (Hatini et al., 1996). (D) RNA *in situ* hybridization analysis of E17.5 mouse embryonic kidneys using markers for nephron progenitors (*Six2*), pretubular aggregates (*Wnt4*), PTs (*Cubn*), podocytes (*Wt1*, *Rhpn1*) and stroma (*Rarb*). Scale bar: 250 μm. Representative examples from *n*=2 biological replicates are shown.

To better characterize the molecular basis for the observed developmental defects, we performed bulk RNA-seq of P0 kidneys. We identified 851 differentially expressed genes (both up- and downregulated) with a q-value<0.05 in *Trim28*^Δ/Δ^ mouse kidneys compared to controls (*Trim28*^f/f^ and *Trim28*^wt/f^) (Fig. 2A; Table S1). Gene Ontology (GO) enrichment analysis of the differentially expressed genes provided insights into the biological processes affected by the loss of TRIM28. Pathways related to innate immune, inflammatory and stress responses, as well as proliferation, were significantly enriched among upregulated genes. In contrast, the downregulated genes were predominantly associated with lipid metabolism and transport functions (Fig. 2B).

Using the Kidney Cell Explorer online tool (Ransick et al., 2019), we found that the top 20 downregulated genes were predominantly expressed in the PT segment. On the other hand, many of the top 20 upregulated genes were normally expressed in various kidney structures with no preference for the PT segment, suggesting involvement of additional nephron segments or a neo-expression in the kidney (Fig. 2C). To further investigate these expression changes on the cellular level, we performed RNA *in situ* hybridization on E17.5 whole embryo sections to visualize the expression of markers relevant for kidney development and/or specific kidney structures (Fig. 2D). *Six2* was expressed in the cap mesenchyme and serves as a marker for the NPC pool. Its expression pattern appeared to be unchanged in *Trim28*^Δ/Δ^ E17.5 kidneys, consistent with the onset of recombination in this transient cell population. Even *Wnt4*, a marker for pre-tubular aggregates, appeared to be unaltered, except in the medulla, where the signal intensity was lower in knockout kidneys. *Cubn*, a marker for the PTs, stained with a generally weaker signal, which again points to PTs as the structures that are predominantly affected in knockout kidneys.

Consistent with the lower expression in bulk RNA-seq, we observed a reduced frequency of *in situ* hybridization signals for the podocyte markers *Wt1* and *Rhpn1*. *Wt1*, which is also expressed in cap mesenchyme, showed a weaker signal specifically in this area, indicating reduced expression in the progenitor cell population. *Rarb*, a marker of the renal stroma, essential for proper organogenesis and maturation, showed more intense staining, suggesting increased gene expression. Higher Rarb expression may affect the known instructive capacity of the kidney stroma on nephron cells (Hatini et al., 1996). Together, these data show that several genes are deregulated in *Trim28*^Δ/Δ^ mouse kidneys, with the downregulated genes strongly associated with the PT segment.

### Deregulation of transposable elements in *Trim28*^Δ/Δ^ kidneys

**Fig. 3.**
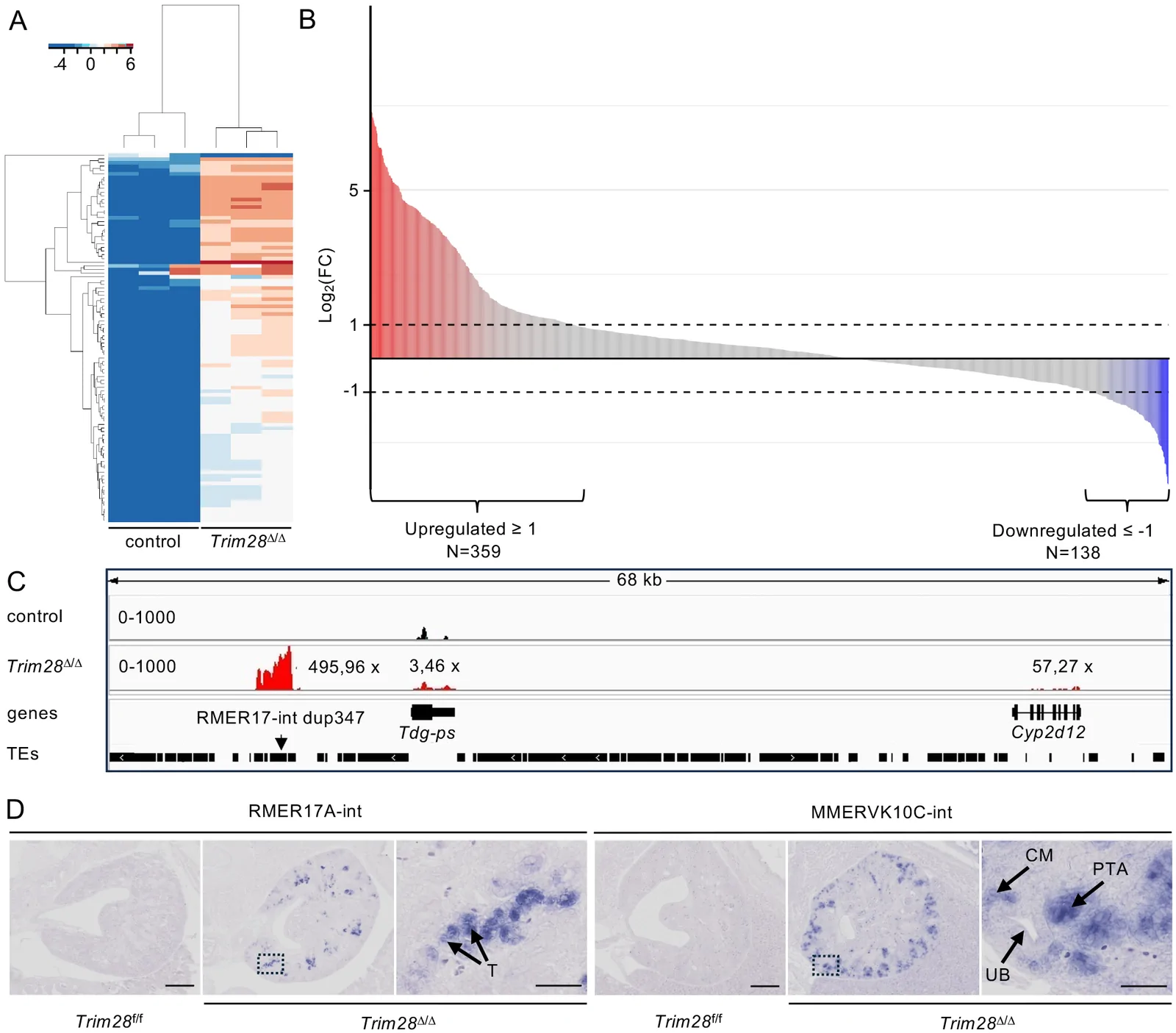
**Differential expression of TEs.** (A) Top 100 differentially expressed transposable element (TE) loci identified from bulk RNA-seq of P0 mouse kidneys. (B) Visualization of all differentially expressed TEs (FPKM≥1 in at least one sample and a variance≥0.5 across all samples in P0 mouse kidneys (*Trim28*^Δ/Δ^ versus control). Expression changes are represented as a color gradient. (C) Genomic localization of the deregulated RMER17-int dup347 and the nearby upregulated *Tdg-ps* and *Cyp2d12* transcripts as visualized by the IGV browser (Robinson et al., 2011). (D) RNA *in situ* hybridization of E17.5 mouse *Trim28*^f/f^ and *Trim28*^Δ/Δ^ kidneys with RMER17-int and MMERVK10C-int probes. Black boxed areas indicate location of regions shown at higher magnification to the right. Black arrows in the magnified regions indicate kidney structures: CM, cap mesenchyme; PTA, pretubular aggregates; T, tubule; UB, ureteric bud. Scale bars: 250 μm; 50 μm (magnification, right). Representative examples from *n*=2 biological replicates are shown for RNA *in situ* hybridization.

*Trim28* plays a crucial role in silencing specific TEs during embryonic development. In addition, it helps maintain proper transcriptional dynamics in early embryos (Rowe et al., 2013). Our bulk RNA-seq results confirmed that several TEs were deregulated, with most of them showing higher expression in *Trim28*^Δ/Δ^ kidneys (Fig. 3A; Table S2). In total, we identified 1400 expressed TE loci in our kidney RNA samples. Of these, 359 (26%) were expressed at higher levels (log_2_FC≥1), while 138 (10%) were expressed at lower levels (log_2_FC≤1) (Fig. 3B). Notably, TE expression changes were not global, but rather affected specific TE families, including MMERVK10C-int, RLTR10B2, RLTR10B, RMER17A-int and MURVY-int (Table S3).

To assess whether these deregulated TEs affected the expression of nearby genes, we analyzed the number of differentially expressed coding and non-coding genes (log_2_ FC≥1) in their vicinity (50 kb up- or downstream of the nearest TE). We found that ∼16% of these upregulated genes had an upregulated TE within a 50 kb range of their gene body, in contrast to only 4% of the downregulated genes (log_2_FC≤1) (Tables S4 and S5). An example is the highly induced TE RMER17A-int, the downstream neighboring genes of which (*Tdg-ps* and *Cyp2d12*) also showed increased expression in *Trim28*^Δ/Δ^ kidneys (Fig. 3C).

To localize the expression of deregulated TE families in tissue sections, we used RNA *in situ* hybridization. While the previously mentioned RMER17A-int was predominantly expressed in well-developed tubular structures, the MMERVK10C-int family was additionally expressed in earlier structures, particularly in the cap mesenchyme (Fig. 3D). These results may not directly correspond to RNA-seq patterns as probes used for *in situ* hybridization may detect entire TE families due to their higher tolerance for mismatches. Nevertheless, these data show that there was localized (tissue or cell type-specific) deregulation of TE families upon *Trim28* deletion. Additionally, the upregulation of a subset of genes may result from the deregulation of nearby TEs in cis.

### Fewer nephron progenitor cells and more proximal tubule cells in developing *Trim28*^Δ/Δ^ kidneys

Our analyses suggested that, during renal development, specific subsets of cells were more affected by *Trim28* deletion than others. We therefore performed single-cell RNA sequencing (scRNA-seq) to investigate cell type-specific expression changes in E18.5 kidneys (*n*=2 males per group). The use of embryonic kidneys rather than P0 mouse kidneys minimizes changes due to birth stress or functional deterioration, and it facilitates more precise timing of the study. Using the 10x Chromium platform, 4527 cells from *Trim28*^Δ/Δ^ kidneys and 6268 cells from *Trim28*^f/f^ kidneys were analyzed, with a median of >30,000 reads and >3500 genes per cell across all samples.

**Fig. 4.**
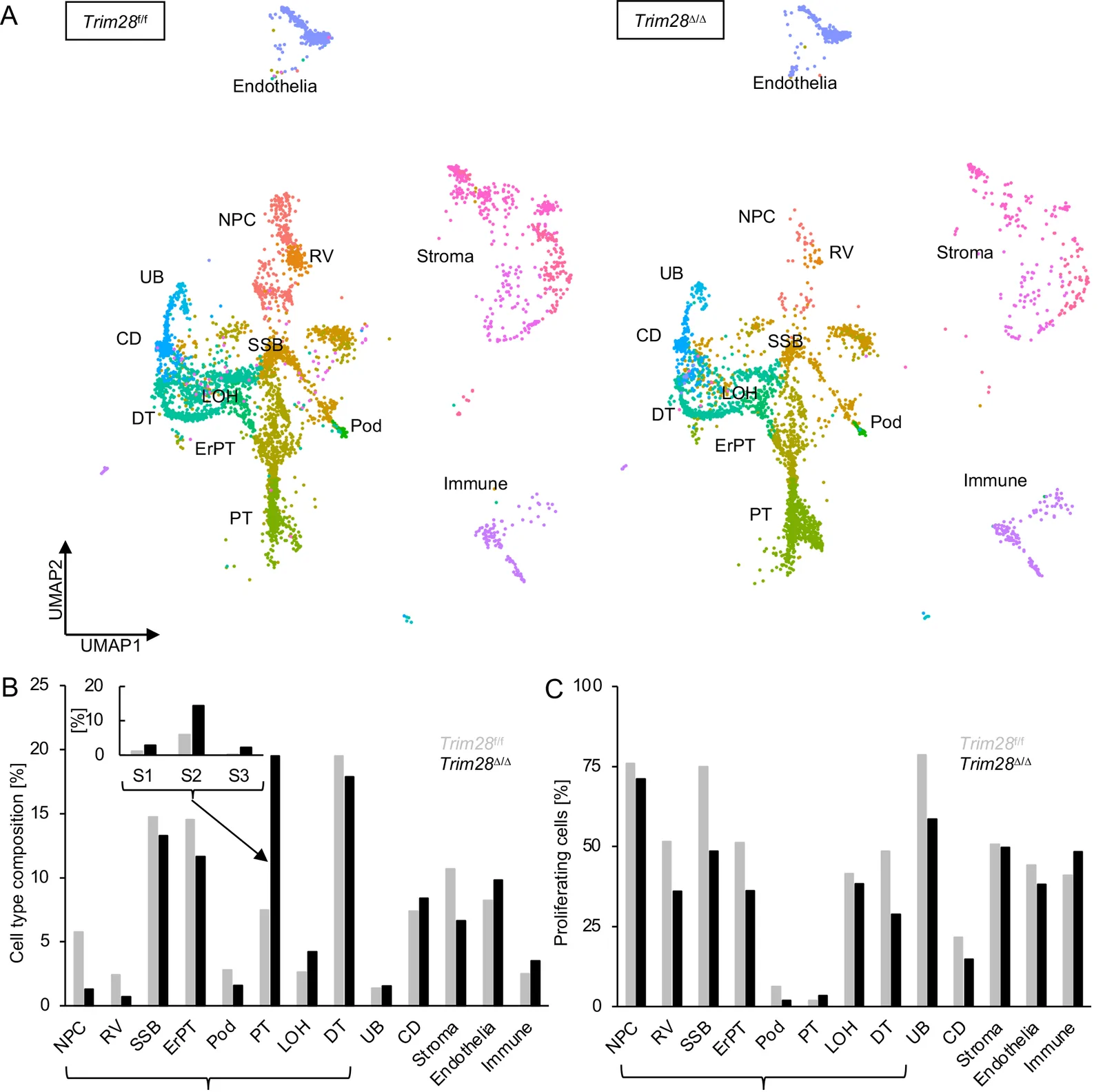
**scRNA-seq analysis of *Trim28* mutant embryonic kidneys.** (A) UMAP representation of the supervised clustering of E18.5 mouse kidney cells into 13 distinguishable clusters. The merged control kidneys are shown on the left, and the conditional knockout embryonic kidneys on the right. The clusters are: nephron progenitors (NPC), renal vesicle (RV), S-shaped body (SSB), early proximal tubule (ErPT), podocyte (Pod), proximal tubule (PT), loop of Henle (LOH), distal tubule (DT), ureteric bud (UB), collecting duct (CD), stroma cells (Stroma), endothelial cells (Endothelia) and immune cells (Immune). (B) Cell type composition of the kidney and subclustering of PT segments S1 to S3 (insert). (C) Proliferation rate per cell type given as percent of cells showing the G2/M and S phase profile. *n*=2 biological replicates per genotype.

Uniform manifold approximation and projection (UMAP) visualization revealed 13 distinct cell clusters in both genotypes. These were identified based on cell-type-specific markers taken from a previous scRNA-seq study of the developing mouse kidney (Combes et al., 2019) (Fig. 4A). A heatmap of the identified clusters clearly illustrates the cell-type-specific expression of these markers (see Fig. S2). A comparative analysis of cell type composition revealed a significant decrease in NPCs and renal vesicle cells in *Trim28*^Δ/Δ^ kidneys (Fig. 4B). This finding is supported by immunofluorescence analyses of Six2^+^ NPCs in E17.5 kidneys (Fig. S3). Interestingly, the reduced frequency of NPCs is accompanied only by a slight decrease in proliferation (Fig. S3; Fig. 4B). In addition to the reduced NPC numbers, more differentiated cell types, such as podocytes, also exhibited a decreased frequency following *Trim28* deletion. Notably, the reduced podocyte frequency was accompanied by a significant reduction in the number of capillaries per glomerulus and a pronounced increase in the capillary cross-sectional lumen area, as well as a slight reduction in glomerular size. Nevertheless, *Trim28* deletion had no effect on the overall structure and integrity of the glomerulus (Fig. S4A-D). Conversely, the populations of PT cells and, to a lesser extent, loop of Henle cells increased (Fig. 4B). Cell cycle analysis based on phase-specific marker gene expression revealed generally reduced proliferation – except for PT cells, which showed a slight increase from a low baseline level (Fig. 4C). Feature plots showed an increased expression of the MMERVK10C-int family throughout the nephrogenic lineage. In contrast, the induction of the RMER17A-int locus was restricted to the PT segment, consistent with the observations from RNA *in situ* hybridization (Fig. S5).

The data demonstrate a strong reduction of NPCs and some descendants, together with an increase in PT cells, combined with a global reduction in the proliferation rate in the nephrogenic lineage, except for PT cells, suggestive of accelerated differentiation.

### Differential gene expression upon TRIM28 loss

**Fig. 5.**
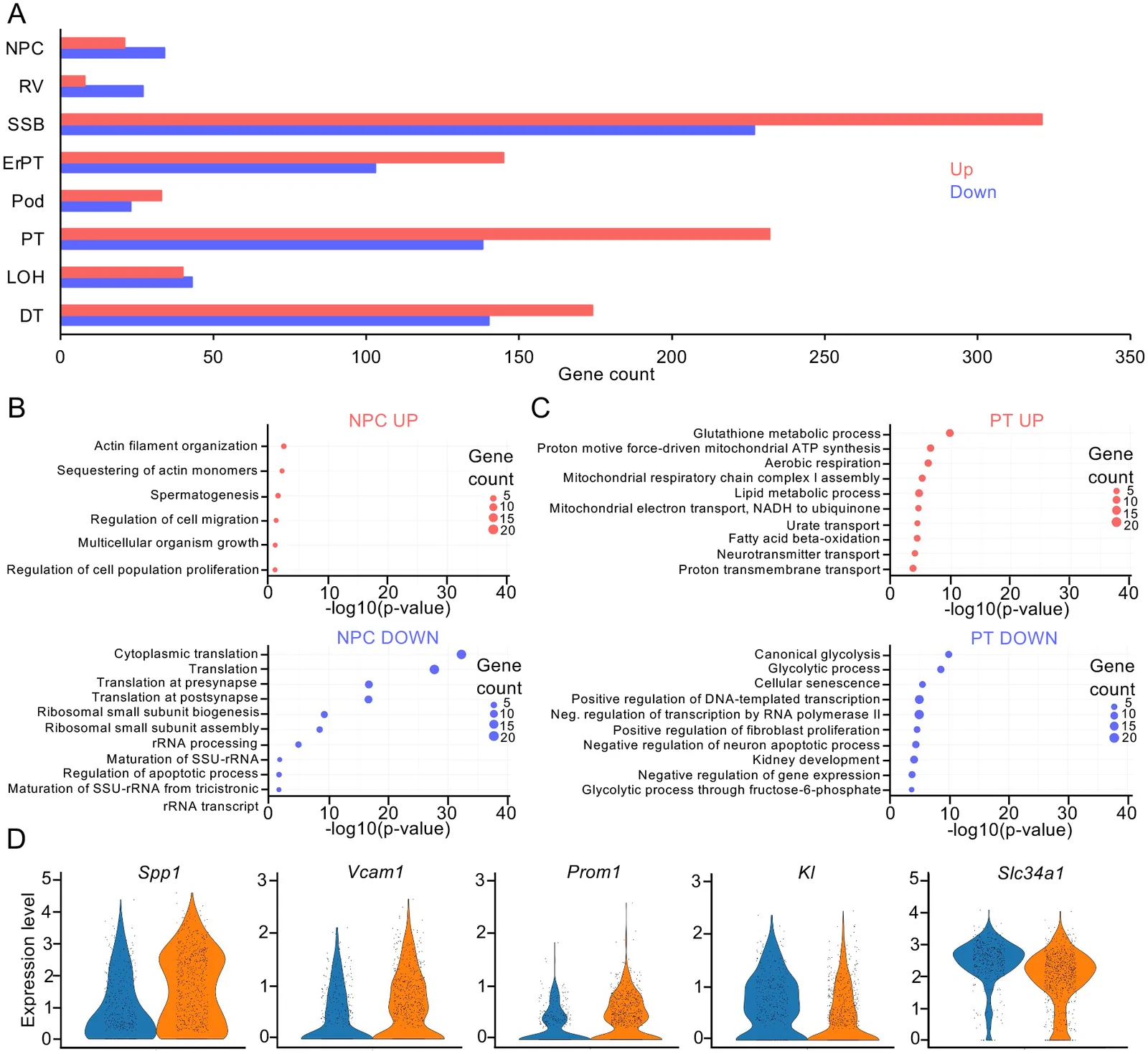
**Transcriptomic changes at single cell level.** (A) Number of differentially expressed genes in distinct cell types of the nephrogenic lineage: nephron progenitors (NPC), renal vesicle (RV), S-shaped body (SSB), early proximal tubule (ErPT), podocyte (Pod), proximal tubule (PT), loop of Henle (LOH), distal tubule (DT). (B,C) GO term analysis of differentially expressed genes in the NPC population (B) and PT cells (C). (D) Violin plot representation of differentially expressed genes in the PT cluster. *n*=2 biological replicates per genotype.

Analysis of differential gene expression in nephrogenic lineage cells revealed widespread changes upon *Trim28* deletion (Fig. 5A). In NPCs, 34 genes were found to be downregulated and 21 to be upregulated. GO term analysis of the downregulated genes revealed a significant enrichment of terms related to translation and ribosome biogenesis (Fig. 5B). In contrast, there was no significant functional enrichment among the upregulated genes and this pattern persisted in renal vesicles (Fig. S6A). Starting with the S-shaped body (SSB) compartment and later in the early proximal tubule (ErPT), upregulated genes were frequently associated with metabolic processes, including mitochondrial respiration and fatty acid β-oxidation, while downregulated genes were often associated with kidney development and glycolysis (Fig. S6B,C). In the strongly affected PT segment, GO term analysis also showed that genes involved in mitochondrial energy and lipid metabolism continued to be upregulated, while genes related to glycolysis, transcriptional regulation, and kidney development were expressed at lower levels (Fig. 5C). The expression changes observed in the other more mature nephron cell types such as podocytes, as well as those found in the loop of Henle and the DT compartments, are also similar to those of PT cells (Fig. S6D,E,F). Of note, the stromal compartment, which is not derived from Six2^+^ NPCs, also exhibited differential gene expression in *Trim28*^Δ/Δ^ kidneys compared to the *Trim28*^f/f^ controls (Fig. S7A-D). This difference was particularly evident in the stromal subpopulation 1, which corresponds to the stroma surrounding the collecting duct. Among the 132 differentially expressed genes, we again found genes associated with glycolytic processes and the response to hypoxia to be downregulated upon *Trim28* loss (Fig. S7A,B).

The PT segment was the most affected in both our histological analysis and our bulk RNA-seq and scRNA-seq analyses. Based on these findings, we hypothesized that these changes could impair the function of this tubular segment, potentially through tubular injury and subsequent inflammation. Consistent with this hypothesis, *Spp1* (also known as osteopontin, and a known proinflammatory cytokine; Jin et al., 2020), *Vcam1* (associated with impaired PT repair and inflammation; Kirita et al., 2020; Melchinger et al., 2024) and *Prom1* (also known as CD133, a marker for injured PT cells; Kusaba et al., 2014) were upregulated. In contrast, *Kl* (klotho), a protective protein in the kidney (Yuan et al., 2022), and *Slc34a1*, a sodium-phosphate cotransporter and terminal differentiation marker (Kusaba et al., 2014), showed significant downregulation (Fig. 5D).

Together, our data suggest that the expression patterns in the PT segment are consistent with ongoing inflammation, potentially associated with cellular stress, incomplete differentiation or repair.

### In *TRIM28* mutant Wilms tumor samples, the loss of TRIM28 leads to reduced protein synthesis in nephron progenitors

Although we did not observe tumor formation in the *Trim28*^Δ/Δ^ kidneys, we explored whether cellular or transcriptional changes resulting from *Trim28* loss might indicate early events or a predisposition toward tumor development.

**Fig. 6.**
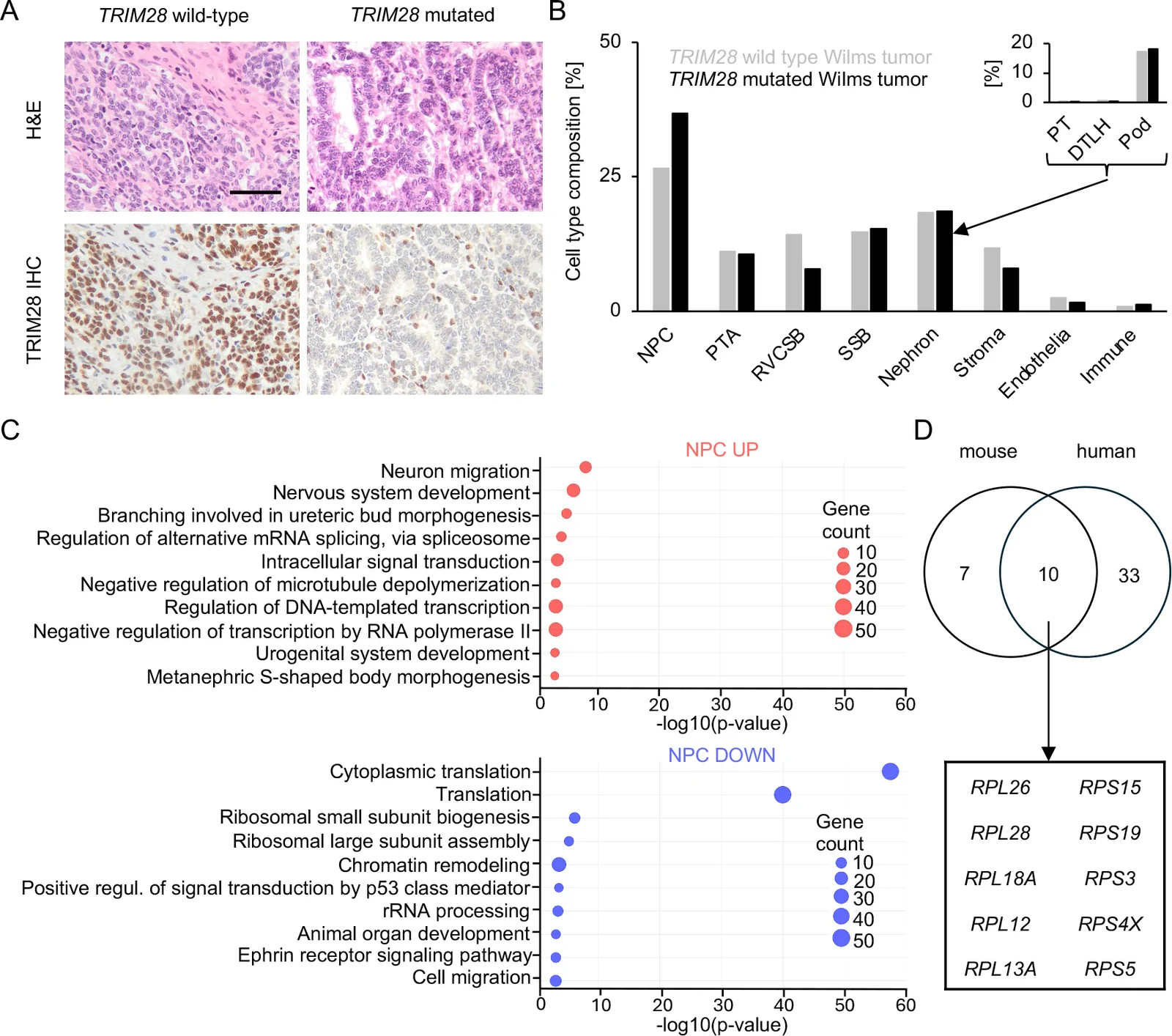
**Comparison of *TRIM28* mutant and *TRIM28* wild-type Wilms tumors.** (A) Comparison of *TRIM28* wild-type and *TRIM28* mutated Wilms tumor (WT) samples, upon H&E staining and immunohistochemistry (IHC) for TRIM28 protein expression. Two representative examples for each tumor type are shown. The full cohort consisted of 126 tumors of which 53 showed a complete TRIM28 loss and 73 were TRIM28^+^. Details on the study are provided in Wegert et al. (2024). Scale bar: 25 μm. (B) Cell type composition of epithelial-type WTs grouped by *TRIM28* mutation status (*n*=2 each) and cell type composition of the nephron (insert). DTLH, combined distal tubule and loop of Henle; NPC, nephron progenitor; Pod, podocyte; PT, proximal tubule; PTA; pretubular aggregates; RVCSB, combined renal vesicles and comma-shaped bodies; SSB, S-shaped body. (C) Gene Ontology (GO) term enrichment analysis of differentially expressed genes in NPCs. (D) Venn diagram of the downregulated ribosomal genes in *Trim28*^Δ/Δ^ mouse kidney and human *TRIM28* mutant tumors, with numbers indicating gene counts and the common ribosomal genes listed below.

To compare our mouse model with the epithelial subtype of WT, we assessed gene expression changes in epithelial-type WTs with either a mutant or wild-type *TRIM28* genotype. There are no morphological features that subdivide the epithelial subtype of WT according to the *TRIM28* mutation status (Fig. S8), even if TRIM28 immunohistochemistry can unequivocally separate these (Wegert et al., 2024) (Fig. 6A). In line with the deregulated expression of TEs observed in *Trim28*^Δ/Δ^ mouse kidneys, *TRIM28* mutant WTs likewise show deregulated TE expression compared to *TRIM28* wild-type WTs (Wegert et al., 2024).

To assess whether *TRIM28* loss generates specific tumor cell populations, we subjected four representative WT samples to snRNA-seq (two *TRIM28* mutant and two *TRIM28* wild-type). This analysis was part of a comprehensive snRNA-seq study of the WT samples, and supervised clustering was performed to identify the different cell types within the tumor samples (A.P. and M.G., unpublished). We did not observe major differences in cell type composition depending on the *TRIM28* status. Interestingly, NPCs even represented the predominant population, while PT cell numbers appeared to be unaffected and very low (Fig. 6B).

The large fraction of NPC in *TRIM28* mutant tumors prompted us to perform GO term analysis of up- and downregulated genes specifically within NPCs. This analysis revealed that downregulated genes were significantly associated with translation-related processes, including cytoplasmic translation and ribosome biogenesis (Fig. 6C). This is remarkably similar to NPC from the *Trim28*^Δ/Δ^ mouse model (see Fig. 5B), with ten downregulated ribosomal genes shared between *Trim28*^Δ/Δ^ kidneys and *TRIM28* mutant tumors (Fig. 6D). This finding highlights a shared transcriptional signature that may point to the mechanism behind the tumor-initiating event.

## DISCUSSION

In this study, we examined the impact of *Trim28* loss on the development of the mouse kidney. We found that homozygous loss of *Trim28* in the nephrogenic lineage resulted in postnatal lethality and a reduced kidney-to-body weight ratio. This reduction in kidney mass may be due to the smaller NPC population that we observed at developmental stages E17.5 and E18.5. The smaller progenitor pool may become exhausted more rapidly and consequently generates fewer nephron structures. However, in contrast to the reduced NPC population size, we observed that the fraction of proliferating NPCs was comparable between *Trim28*^Δ/Δ^ and *Trim28*^f/f^ animals at these later developmental stages. A previous report (Short et al., 2014) demonstrated that NPC proliferation is strongly stage-dependent, with higher proliferation rates at earlier developmental stages (e.g. E13.25) than at later stages (e.g. E17.25). Therefore, it is possible that NPC proliferation is impaired at earlier developmental time points in *Trim28*^Δ/Δ^ animals compared to *Trim28*^f/f^ controls. Future studies should therefore include earlier developmental stages in order to fully assess the impact of *Trim28* loss on NPC proliferation dynamics.

Reduced NPC frequencies have previously been observed in mice with targeted mutations of factors that are involved in the balancing of NPC self-renewal and differentiation. These factors include members of key signaling pathways such as the BMP, FGF and WNT pathways (Carroll et al., 2005; Karner et al., 2011; Barak et al., 2012; Tomita et al., 2013). Whereas *Bmp7*-deficient mice show a moderate loss of NPCs, which can no longer differentiate, mutations in *Fgf9* and *Fgf20* are associated with a dose- and time-dependent loss of NPCs. A more severe phenotype has been described in mice with targeted *Wnt9b* deletion. These mice exhibit disturbed cell cycling and nephron induction, as well as a rapid loss of NPCs. The extent to which TRIM28 interacts with these pathways is an interesting question that should be the subject of future investigations.

The Six2-cre transgene alone has previously been reported to lead to reduced kidney size when expressed in the CD1 background (Perl et al., 2024). However, in line with recent findings (Xue et al., 2025), we did not observe any reduction in kidney mass in our Six2-cre transgene mice that were bred on the C57BL/6 background. This suggests that the effect of the Six2-cre transgene is background-dependent. Thus, the reduction in kidney size observed here is solely due to the absence of the TRIM28 protein.

In addition to the reduced NPC population, our analyses revealed severe structural and functional impairment, specifically of the PT compartment in *Trim28*^Δ/Δ^ kidneys. This includes tubular disorganization and reduced luminal space, which is a possible cause of the observed lethality in these mice (Curthoys and Moe, 2014). Although the reduced number of glomerular capillaries and the pronounced increase in capillary cross-sectional lumen area are most likely not lethal by themselves, they do add complexity to the phenotype of *Trim28*^Δ/Δ^ kidneys. Consequently, the precise cause of death of the animals is not fully understood. Besides the structural disorganization, our single cell transcriptomic analyses show an increased relative proportion of PT cells, indicative of a disrupted balance between nephric cell populations. It is tempting to speculate that *Trim28*^Δ/Δ^ NPCs have an increased propensity to differentiate along the PT lineage, thereby contributing to the reduced NPC fraction and the increase in the PT cell population. Alternatively, the reduction in NPC and the increase in PT frequencies could be two independent processes. The extent to which the shift towards the PT compartment could affect other cell populations in the kidney beyond their relative reduction is unknown. In this context, it is interesting to note that we found *Wnt4* expression to be strongly reduced in the medulla upon *Trim28* deletion. As *Wnt4* is expressed by medullary stromal cells that do not originate from the Six2^+^ NPC compartment, an indirect effect emanating from the tubular compartment may lead to the loss of *Wnt4* expression in the medulla.

The PT segment of the nephron is a primary site of kidney injury due to its significant energy demands for solute transport and its reliance on oxidative metabolism (Schaub et al., 2021). Decreased expression of glycolysis-related genes in PT cells in combination with an increase in genes that are associated with lipid and fatty acid metabolism may indicate metabolic reprogramming and an increased energy demand during kidney damage (Eymael et al., 2023). The concomitant upregulation of genes associated with glutathione metabolism suggests enhanced antioxidant activity in response to ongoing oxidative stress (Lash, 2024). Our single-cell data show that *Spp1* and *Vcam1*, genes involved in inflammation (Jin et al., 2020; Nie et al., 2022), are upregulated in the *Trim28*^Δ/Δ^ PT segment (Buse et al., 2024; Melchinger et al., 2024), which is consistent with our observations from bulk RNA sequencing, where an increased expression of inflammation- or innate immunity-associated genes was observed. Likewise, the upregulation of *Prom1* is in support of ongoing repair processes, as this gene is known to be induced upon kidney injury (Kusaba et al., 2014) and during subsequent regeneration (Angelotti et al., 2012). Finally, *Kl*, which has previously been described as a renoprotective marker in different models of acute kidney injury, is downregulated (Hu et al., 2010). It is also worth noting that we found expression changes in the stromal compartment that point to glycolytic and hypoxia-associated processes which further supports the idea that the genetic change in the tubular compartment has an influence on stromal cell populations. Taken together, these findings suggest that, following the deletion of *Trim28*, the PT segment is subjected to considerable stress that is potentially induced by inflammation and may render it non-functional. This defect, in conjunction with the additional defects in the tubular and glomerular systems described in this study, may account for the early lethality of *Trim28*^Δ/Δ^ animals. However, as this conclusion is based solely on structural abnormalities and transcriptomic alterations rather than direct physiological measurements, future studies should incorporate *in vivo* functional assays to substantiate this hypothesis.

The extent to which the deregulation of TEs in *Trim28*^Δ/Δ^ kidneys plays a role in these pathological processes is not yet known. Interestingly, the deletion of the DNA methyltransferase 1 (*Dnmt1*) in the developing kidney has previously been shown to result in reduced kidney mass, increased expression of endogenous retroviral elements and a concomitant upregulation of inflammatory genes (Li et al., 2019; Wanner et al., 2019). The deregulation of the MMERVK10C-int family, which was found to be one of the most prominently upregulated TEs in our bulk sequencing analyses, is of particular interest in this context, as it has previously been shown to be deregulated upon *Trim28* loss in neural progenitor cells and murine embryonic stem cells (Fasching et al., 2015; Asimi et al., 2022). Furthermore, it has been shown to cause inflammation in the developing brain (Jönsson et al., 2021). Together, these findings are in support of the notion that a TE-triggered inflammatory response contributes to the developmental defects upon *Trim28* loss.

Our single-cell analyses revealed that the MMERVK10C-int family was predominantly expressed in early nephron structures and some developed tubular segments. By contrast, the expression of the RMER17A-int family was limited to the PT segment. These findings suggest that *Trim28* plays a role in the cell-type- and differentiation-specific silencing of TEs in different nephron cell populations. The cell type-specific TE deregulation reported here is consistent with the role of TRIM28 and KRAB-ZFPs in enforcing epigenetic silencing of TEs during differentiation (Turelli et al., 2014). In addition to the silencing of TEs, there is an increasing recognition of their role as a prolific source of cis-regulatory elements, including enhancers and transcription factor binding sites, which are co-opted for the regulation of host genes (Chuong et al., 2017). This suggests that the deregulation of TEs upon TRIM28 loss may have broader consequences for the transcriptional identity of the developing kidney. The extent to which different TEs may contribute to an inflammatory response in different cellular compartments remains unknown. Of note, although we also observed differential TE upregulation in human TRIM28-negative tumor samples, we did not observe an inflammatory gene signature in these tumors and WTs are generally considered to be immunologically cold (Palmisani et al., 2021). Several studies suggest that the tumors may suppress or evade the immune system through various mechanisms, e.g., by increasing the expression of DNA repair genes (Higgs et al., 2022). Whether these mechanisms also play a role in TRIM28-negative tumors remains to be determined and should be the subject of further analysis.

In addition to potentially triggering the expression of inflammatory genes in response to aberrant TE expression, the de-repression of TE loci upon *Trim28* loss may also affect cis-acting activators contained within these loci. This mechanism has previously been shown to be important in preventing the untimely activation of genes in the vicinity of TRIM28-repressed TE loci during early development (Rowe et al., 2013). Our own global gene expression analyses show that some of the differentially upregulated coding and non-coding genes in *Trim28*^Δ/Δ^ kidneys are indeed located in proximity to active TE loci. However, the number of genes with higher expression was rather low, and none of these genes has yet been shown to play a role in kidney development. Therefore, the extent to which these genes contribute to the observed phenotype needs to be addressed in future studies.

In addition to the genes that were differentially upregulated upon *Trim28* deletion, we also observed that several genes within different cellular compartments were expressed at lower levels. Specifically, the lower expression of ribosomal genes within the NPC compartment is potentially relevant, as we also observed a similar downregulation of such genes in tumor-associated TRIM28-negative NPCs. While this interesting commonality was observed in mouse and tumor NPCs, no tumor formation was observed in the Six2-cre transgenic animals, most likely due to early lethality. The differential expression of ribosomal genes has, however, previously been reported in connection with several tumor entities (El Khoury and Nasr, 2021). In particular, the reduced expression of the *RPS5* ribosomal subunit, which we observed in both mouse NPCs and tumor-associated NPCs, is of potential relevance. Specifically, *RPS5* downregulation was found to be part of a gene signature associated with a higher risk of tumor recurrence and tumor progression in colon cancer (Bandres et al., 2007). Moreover, the *RPS5*-interacting subunit *RPS7* has previously been reported to be expressed at low levels in colorectal cancers, and its overexpression has been shown to inhibit cancer growth (Zhang et al., 2016).

The lack of tumor formation in our mouse model is currently not explained. To model WT formation in the mouse, inducible systems such as the Cited1-CreERT2 system could be instrumental in the future. Such a system can induce mosaic inactivation of *Trim28* within the nephrogenic progenitor population and reduce the detrimental effect on kidney development to allow for longer survival of mutant clones and tumor formation to occur. The aforementioned strategies target the fate committed mesenchymal progenitor cell compartment, which gives rise to the tubular epithelial cell types of the nephron through mesenchymal-to-epithelial transition (MET). As it is not known whether the tumor-initiating cell in *TRIM28* mutant WTs derives from a progenitor cell type that arises before or after MET, it may also be interesting to target the developing nephron at a post-MET stage. In this regard, the Ksp1.3/Cre (Chd16-Cre) system (Shao et al., 2002) could be instrumental, as it enables the specific deletion of target genes in tubular epithelial cells in the developing kidney. Furthermore, the recent development of robust human induced pluripotent stem cell (hiPSC)-NPC expansion protocols, both in 2D (Huang et al., 2024) and via 3D aggregate-based CFY medium (Araoka et al., 2025), opens the possibility of modeling *TRIM28* loss in human NPC systems, which could provide complementary insights into the tumor-initiating mechanisms. It may also help to unravel the puzzling observation that NPCs are abundant in human *TRIM28*-mutant WT but depleted in our mouse model. This in turn suggests that accelerated differentiation and stem cell depletion in the mouse may be decisive factors.

### Study limitations

One limitation of this study is that only two biological replicates were used per genotype in the scRNA-seq analyses. Future studies would benefit from including a higher number of biological replicates to increase statistical power. Additionally, while our data show that *Trim28*^Δ/Δ^ NPC frequencies decreased and PT cell frequencies increased, we currently do not have an explanation for the underlying mechanisms that led to these results. Our data indicate that the reduced NPC number is at least not fully explained by reduced proliferation. Although we cannot exclude apoptotic loss of *Trim28*^Δ/Δ^ NPCs, a plausible explanation for the different NPC and PT cell numbers is a combination of accelerated differentiation and reduced stem cell maintenance. Future studies clarifying this question will require genetic lineage tracing, the gold standard for such tests.

## MATERIALS AND METHODS

### Animal experiments

Six2-cre mice [Tg(*Six2*-EGFP/cre)1Amc/J] (RRID: IMSR_JAX:009606; Kobayashi et al., 2008) were obtained from C. Englert (Fritz Lipmann Institute, Germany). The *Trim28*^tm1.1Ipc^ (*Trim28*^f^) (Cammas et al., 2000; RRID: IMSR_JAX:018552) mouse line was obtained from D. Trono (EPFL, Switzerland). All animal experiments were approved by the relevant local authorities (Az.: RUF-55.2.2-2532-2-783-16) and conducted in accordance with the guidelines of the German Animal Welfare Act (Tierschutzgesetz).

The Six2-cre allele was genotyped by genomic PCR using three primers to amplify the *Six2* (281 bp) and Six2-cre (209 bp) allele (Table S6). Similarly, the *Trim28*^f^ allele was genotyped using three primers (Table S6), yielding distinct fragments for the *Trim28* wild-type (156 bp), the *Trim28*^f^ (256 bp) and the *Trim28*^Δ^ (275 bp) alleles.

### Human material

Frozen WT and corresponding kidney tissues were collected under informed consent as part of the pathology reference review and biobanking process within the SIOP2001/GPOH studies. Ethical approval was granted by the Ethikkommission der Ärztekammer des Saarlandes (reference numbers 23.4.93/Ls, 136/01 and 248/13) and Medizinische Ethikkommission an der JMU Würzburg (336/21_z-sc).

### Histopathology and immunohistochemistry

Euthanized mouse embryos or dissected kidneys were fixed in 4% paraformaldehyde-PBS overnight at 4°C, embedded in paraffin and sectioned at 5 μm using a HM 340E Rotary Microtome (ThermoFisher Scientific), followed by Hematoxylin and Eosin (H&E) staining.

For immunohistochemistry, paraffin sections were deparaffinized, rehydrated and subjected to heat-induced antigen retrieval in 10 mM sodium citrate buffer (pH 6.0), 0.05% Tween-20. Sections were blocked in 0.2% gelatine/PBS for 1 h at room temperature and incubated overnight at 4°C with primary antibodies. The next day, sections were washed with PBST (PBS with 0.05% Tween-20), incubated with fluorophore-conjugated secondary antibodies (1:200) and washed again. Nuclei were counterstained with Hoechst 33342 (1:200 in PBS, Thermo Fisher Scientific, H3570). Primary antibodies were: rabbit-anti-TRIM28 (1:1000, Sigma-Aldrich, HPA064033), rabbit-anti-E-Cadherin (1:200, Sigma-Aldrich, SAB5700789), mouse-anti-Megalin (1:500, Santa Cruz Biotechnology, sc-515772). Secondary antibodies were: Alexa 488-goat-anti-rabbit (1:200, Invitrogen, A-11088) and goat-anti-mouse Cy3 (1:200, Merck, AP130C). Imaging was performed using a Nikon Eclipse Ti confocal microscope and NIS-Elements AR 3.2 software. Alternatively, slides were scanned at 40× resolution using the Olympus Slide Scanner (VS120 L100) and analyzed with QuPath (version 0.5.1). Immunohistochemical analysis of FFPE human tissue was performed following the protocol described in Wegert et al. (2024).

### Quantitative real-time PCR

qRT-PCR was performed at an annealing temperature of 60°C using SYBR Green (Merck; 1:2000 in H_2_O/0.5% DMSO). *Trim28* (exons 3-4) and *Hprt* (used as the reference gene) were amplified using primers listed in Table S6. Specificity was confirmed by melting curve analysis. Experiments were performed in biological triplicates with technical triplicates for each sample using a CFX Opus 96 System (Bio-Rad).

### RNA *in situ* hybridization

Sections of paraffin-embedded embryos were hybridized with digoxigenin-labeled antisense riboprobes as described previously (Leimeister et al., 1998). Partial cDNAs for *Six2*, *Wt1*, *Rarb*, *Cubn* and *Rhpn1* were amplified from mouse cDNA and cloned into pCS2p vectors to generate templates for antisense RNA transcription. For primers used to amplify the cDNAs, see Table S6. The *Wnt4* riboprobe was kindly provided by A. Kispert (Kispert et al., 1998).

### Transcriptome sequencing and data analysis

Total RNA was isolated from flash frozen kidney tissue using TRIzol reagent (Invitrogen, Thermo Fisher Scientific, 15596026) and the concentration was measured using an Invitrogen Qubit Fluorometer. Total RNA-seq was performed by Novogene Corporation Inc (150 bp paired-end, average of 57.6 mio reads). Initial quality assessment was conducted using FastQC version 0.11.8 (https://www.bioinformatics.babraham.ac.uk/projects/fastqc/). Adapter and read trimming were carried out using TrimGalore version 0.6.1 (https://www.bioinformatics.babraham.ac.uk/projects/trim_galore/), utilizing Cutadapt version 2.3 (Martin, 2011). The trimmed reads were then mapped to the ENSEMBL mouse reference genome GRCm38.99 using STAR version 2.5.4b.

For downstream processing, samtools v1.3 (Li and Durbin, 2009), along with htslib v1.3 (Li, 2011), was used for sam-to-bam conversion, sorting and indexing of the alignment files. Gene annotation was carried out using the ENSEMBL mouse GRCm38.99 annotation, with the addition of Transposable Elements. FPKM-values and differential expression were calculated using the Cufflinks package, version 2.2.1 (Trapnell et al., 2010).

Principal component analysis (PCA) was conducted on all samples using log2-transformed FPKM values for genes that were expressed in more than one sample (FPKM≥1) and had a coefficient of variation greater than or equal to 0.5. The PCA was carried out using the prcomp function from the R stats package (v3.4.4). Following this, the 100 genes with the highest principal component loadings (i.e. the highest absolute correlation coefficients with one of the first three principal components) were selected. Unsupervised complete linkage clustering was then performed on both the rows and columns, using the Euclidean distance metric, and visualized with the heatmap.2 function from the gplots R package (v3.0.1).

### scRNA-seq

Pregnant female mice were euthanized on E18.5 by CO_2_ inhalation followed by cervical dislocation. After laparotomy, embryos were harvested, euthanized, and kidneys were dissected. To minimize stress-induced transcriptional artifacts, a cold dissociation protocol was applied using *Bacillus licheniformis* cold-active protease (CAP), as previously described (Ransick et al., 2019).

Single-cell suspensions were processed using the Chromium Next GEM Single Cell 3′ Reagent Kits v3.1 (Dual Index) with Feature Barcode technology for cell multiplexing, according to the manufacturer's instructions (10x Genomics, CG000390 Rev B). Cells from different mice were labeled with cell multiplexing oligonucleotides (CMOs) following the protocol (10x Genomics, CG000391) and loaded onto the Chromium Controller for Gel Beads-in-Emulsion (GEM) generation. Reverse transcription and barcoding were carried out within the GEMs. Aiming for a cell concentration of 1400 cells/μl, ∼40,000 cells were loaded, resulting in a final recovery of ∼24,000 cells. After cDNA amplification, separate libraries were prepared for gene expression and CMOs. Libraries were pooled and sequenced on a NovaSeq 6000 S2 flow cell (Illumina).

### Workflow for scRNA-seq analysis

Raw sequencing data were processed using the cellRanger v6.0.0 (10x Genomics) workflow. Downstream quality control and analysis were carried out in R v4.1.0 using Seurat v4.0. Cells were filtered based on standard quality metrics: those with fewer than 200 detected genes or more than 10% mitochondrial gene content were excluded.

Gene expression values were normalized using SCTransform (Seurat), followed by PCA for dimensionality reduction. UMAP was applied for visualization. Louvain graph-based clustering was used to define transcriptionally distinct cell populations.

Cell-type annotation was performed by projection onto a reference kidney single-cell map derived from publicly available mouse data (Combes et al., 2019). Differential gene expression analysis was conducted using the Seurat FindMarkers function based on the Wilcoxon rank-sum test with Bonferroni correction. Genes with a log_2_ fold change >0.25 and an adjusted *P*-value <0.05 were considered significant.

GO enrichment of differentially expressed genes was performed to identify biological processes associated with specific cell populations. Cell cycle analysis was carried out using the Seurat CellCycleScoring function based on canonical S and G2/M phase marker sets. Proliferation indices were calculated as the proportion of nuclei in either the S or G2/M phase. All statistical analyses and visualizations were performed in R, and *P*-values <0.05 were considered statistically significant.

### Statistical analysis

Statistical comparisons were performed using two-sided Welch's *t*-tests. For multiple comparisons in Fig. 1D, *P*-values were adjusted using the Benjamini-Hochberg procedure. Welch's *t*-test was chosen to account for unequal group sizes and variances between genotypes. For qRT-PCR experiments (Fig. S1), three biological replicates were measured in technical triplicates. Details on statistical tests used for analyses presented in the supplement are given in the Supplementary materials and methods. Data are presented as mean±standard error of the mean (s.e.m.).

## Supplementary Material



10.1242/develop.205188_sup1 Supplementary information

Table S1. Differentially expressed genes between knockout and control samples from bulk RNA sequencing

Table S2.Top 100 differentially expressed transposable elements identified between knockout and control samples

Table S3. Deregulated transposable element families

Table S4. List of upregulated genes with upregulated transposable elements within a 50 kb distance in knockout samples

Table S5. List of downregulated genes with upregulated transposable elements within a 50 kb distance in knockout samples

Table S6. List of primers used in this study
