# Supplementary material for: The absence of Trim28 in nephron progenitors results in impaired kidney development and function
Source: Development. 2026 Aug 13;153(15):dev205188. doi: 10.1242/dev.205188 (PMC13535099; doi:10.1242/dev.205188)
Supplement: Supplementary file 1 [file develop-153-205188-s1.pdf]

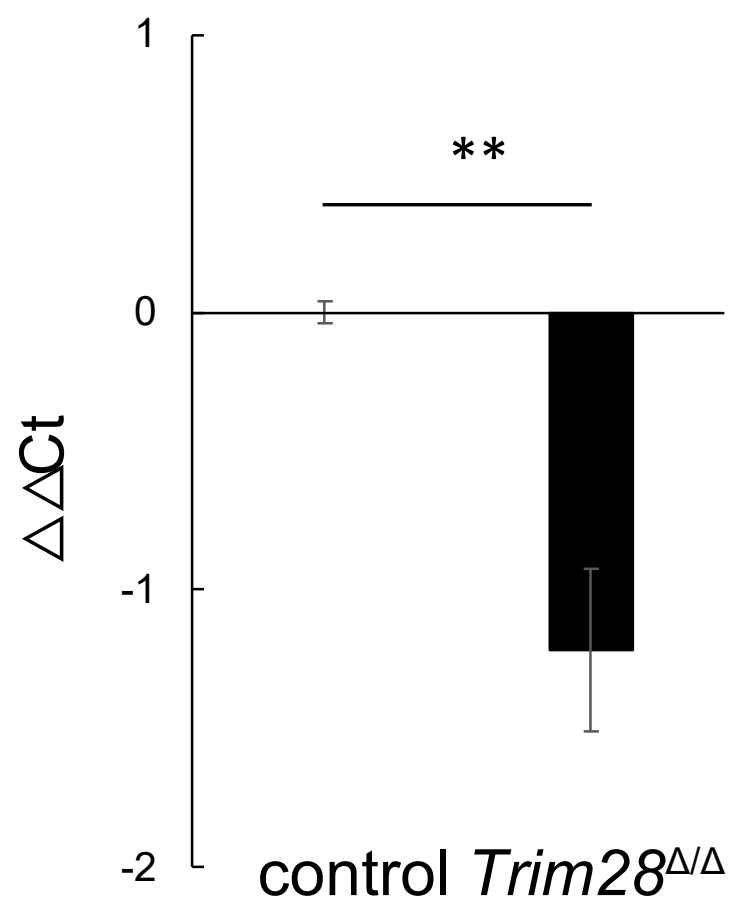

Fig. S1. Quantitative analysis of *Trim28* expression in mouse kidneys

Quantitative RT-PCR analysis of *Trim28* mRNA in embryonic day 18.5 (E18.5) *Trim28*$^{\Delta/\Delta}$ (*n*=3) and control RNA (*n*=3). Data represent mean ± s.e.m. of three biological replicates (each measured in technical triplicates). Statistical significance was determined by an unpaired two-tailed Welch's t-test. **P < 0.01

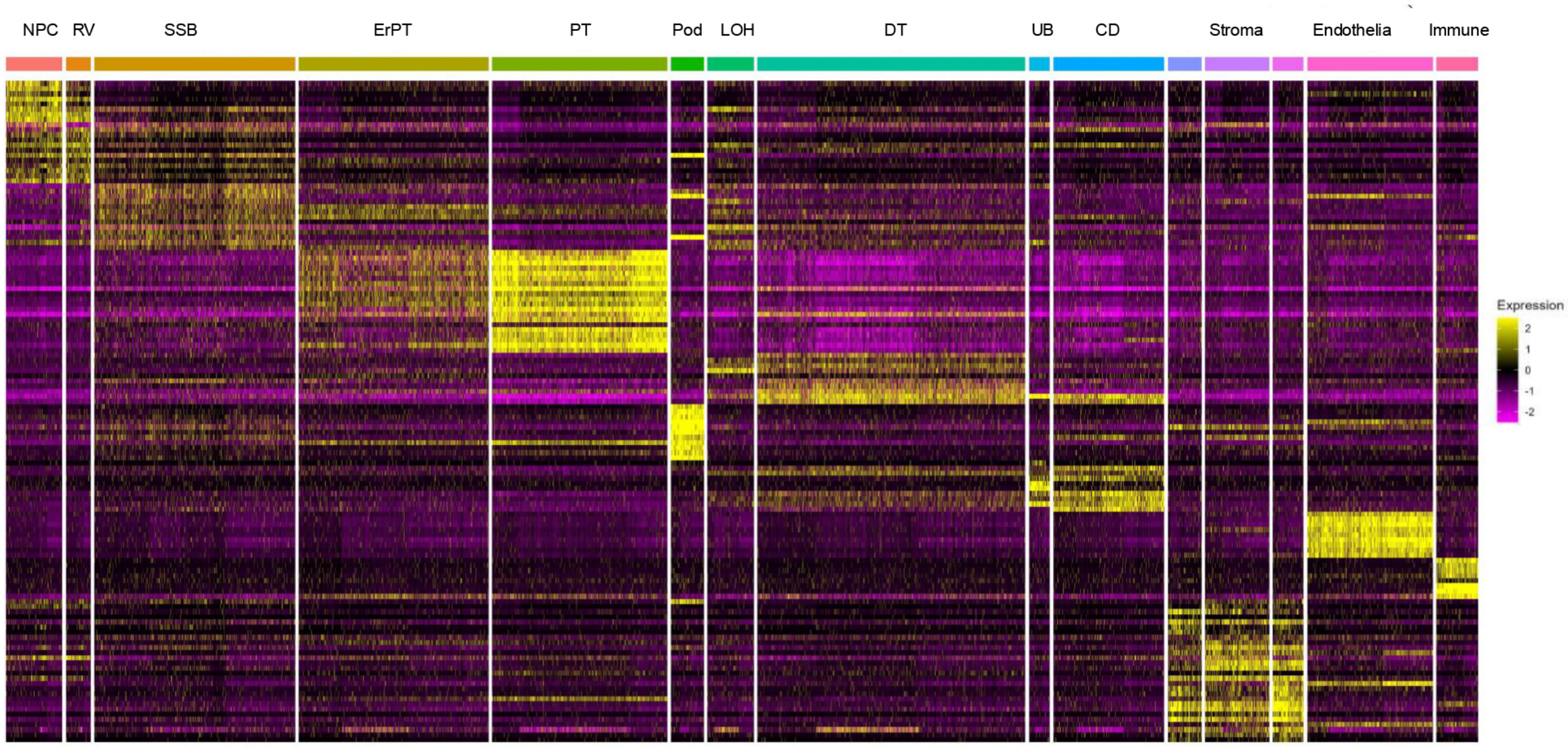

Fig. S2. Marker gene expression in distinct cell populations

Heatmap of the markers used to identify the different cell types (NPC – nephron progenitor; RV – renal vesicle; SSB – S-shaped body; ErPT – early proximal tubule; Pod – podocytes; PT – proximal tubule; LOH – loop of Henle; DT – distal tubule; UB – ureteric bud; CD – collecting duct; Stroma – stromal cells; Endothelia – endothelial cells; Immune – immune cells). Embryonic day 18.5 (E18.5) *Trim28*$^{\Delta/\Delta}$ (*n*=2) and control RNA (*n*=2).

Development • Supplementary information

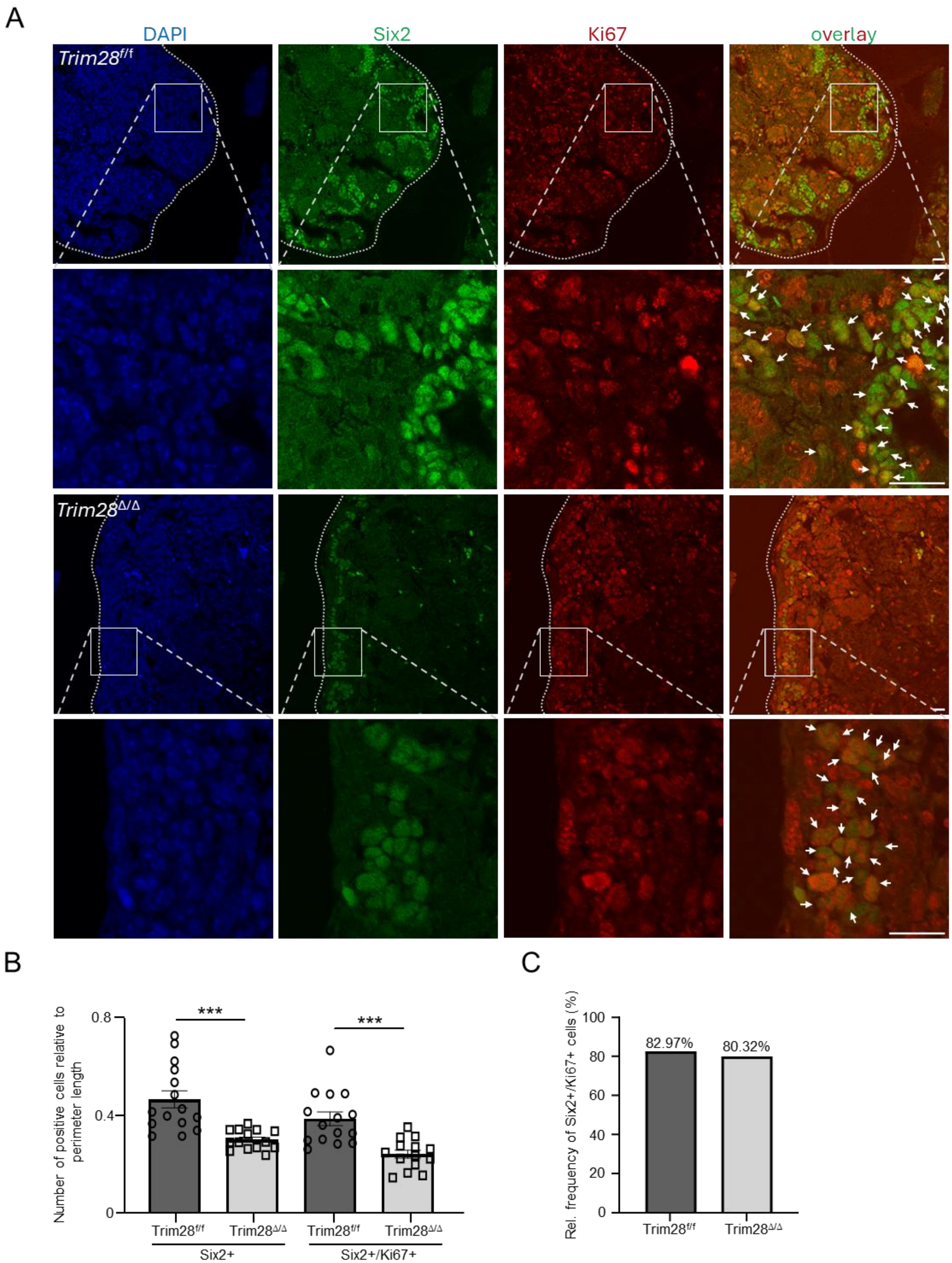

Development • Supplementary information

Fig. S3. Number of Six2+ NPCs is reduced, while the relative frequency of cells in cell cycle is only slightly reduced in E17.5 *Trim28*$^{\Delta/\Delta}$ kidneys.

(A) Representative confocal microscopy images showing fields of DAPI, Six2, Ki67 stained sections of E17.5 *Trim28*$^{f/f}$ and *Trim28*$^{\Delta/\Delta}$ kidneys. White rectangles indicate regions shown at higher magnification below. Dotted lines indicate kidney perimeters. White arrows in the overlay picture indicate Six2/Ki67 double positive cells. Scale bars: 20 µm. **(B)** Quantitation of average numbers of Six2 positive (Six2+) and Six2/ Ki67double positive (Six2+, Ki67+) cells relative to length of perimeter. **(C)** Relative frequency of proliferating (Six2+, Ki67+) cells.Number of biological replicates per genotype: *n*=3, error bars indicate s.e.m., ***P< 0.001

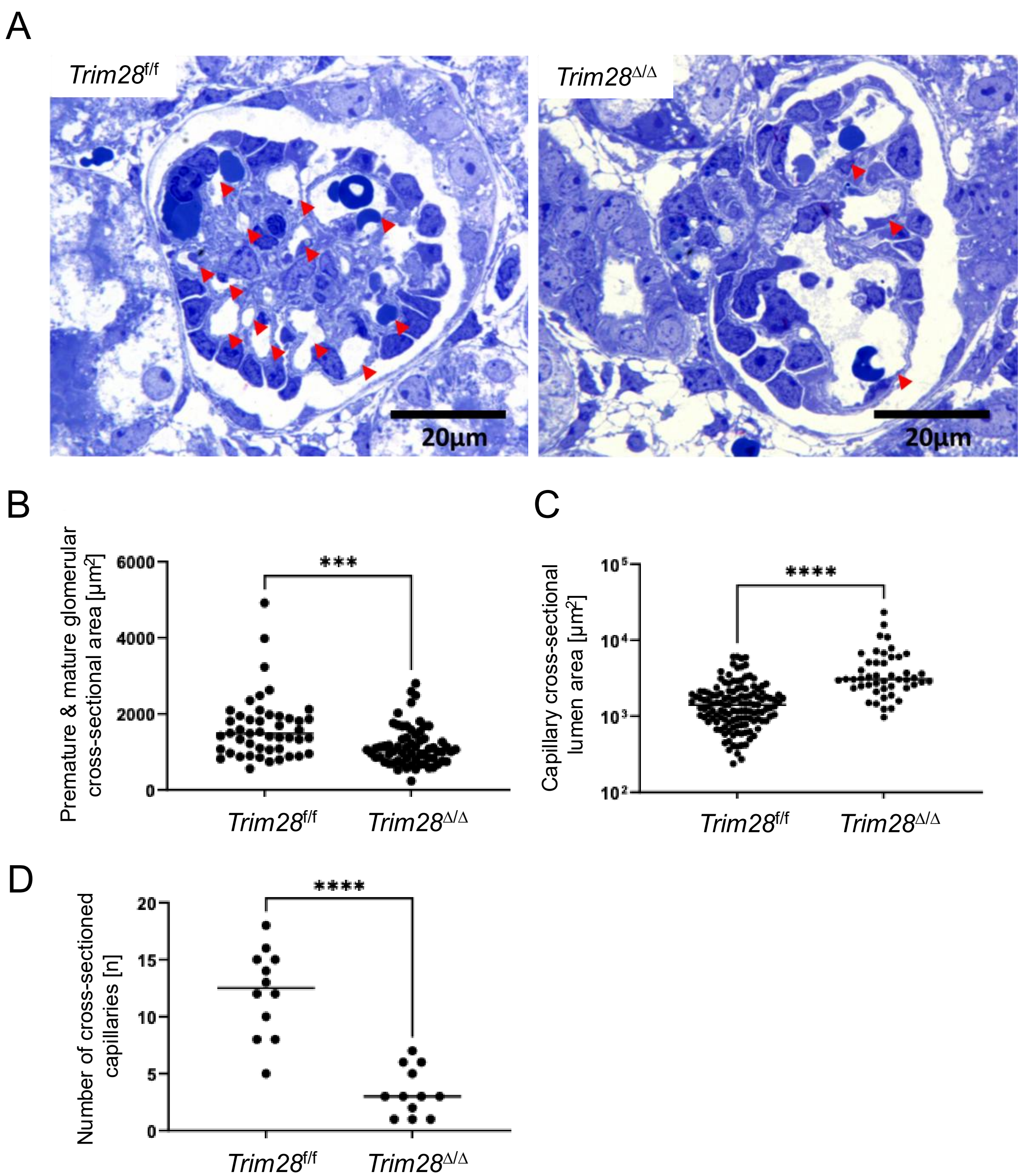

Fig. S4. Altered capillary formation in glomeruli from *Trim28*$^{\Delta/\Delta}$ mice

(A) Representative examples of toluidine-stained semi-thin sections showing a glomerulus for each genotype. Red arrowheads indicate glomerular capillaries. Quantification of **(B)** glomerular cross-sectional area of premature and mature glomeruli, **(C)** capillary cross-sectional lumen area and **(D)** the numbers of cross-sectioned capillaries per glomerulus were evaluated by manual tracing of capillary lumina and glomerular tuft area using Zen software (Zeiss, Oberkochen, Germany). Significant differences are indicated by ****P*=0.0001, *****P*<0.0001 as assessed by Mann-Whitney test. Postnatal day 0 (P0) *Trim28*$^{\Delta/\Delta}$ (*n*=3) and control kidneys (*n*=2).

Development • Supplementary information

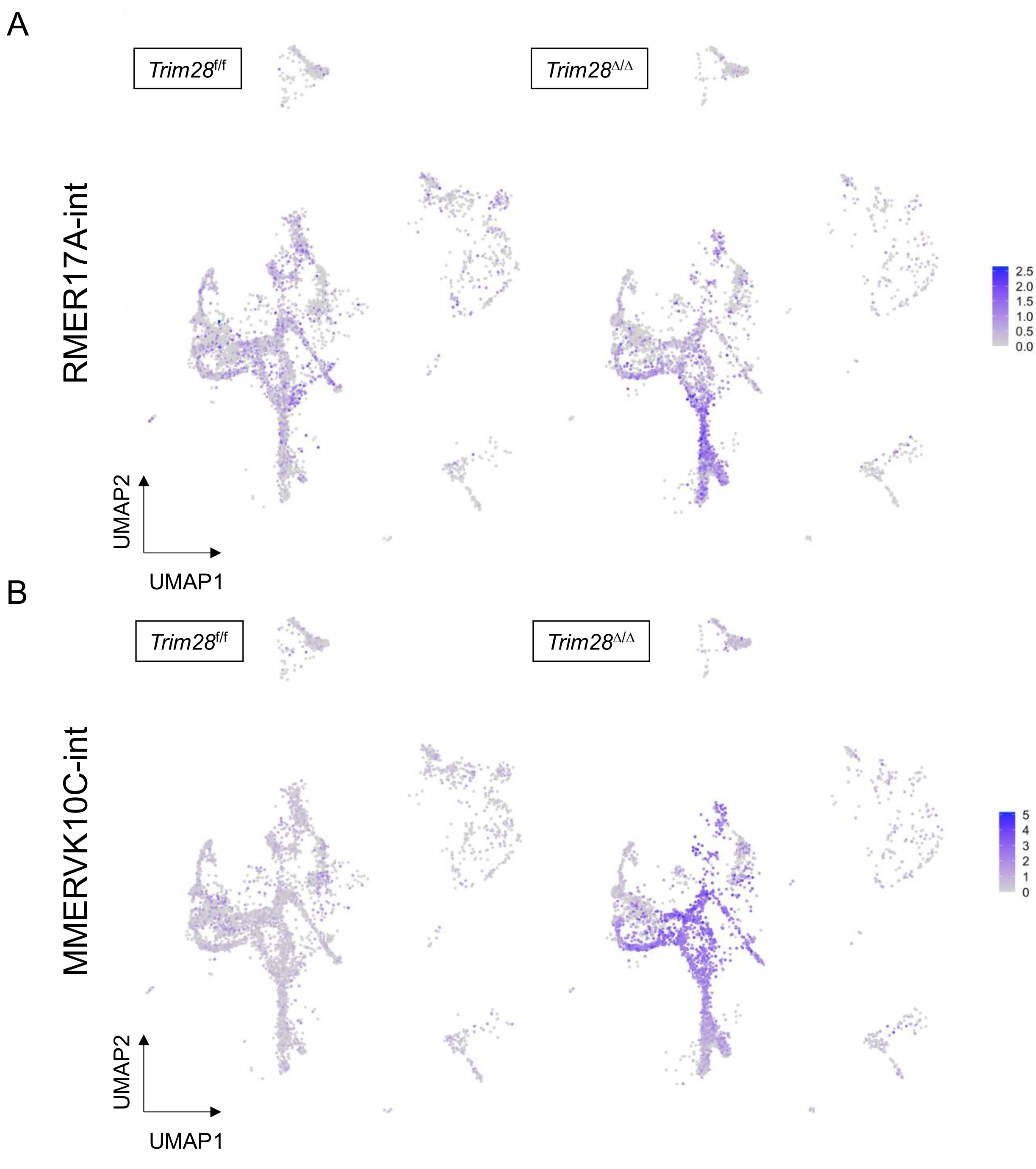

Fig. S5. Comparative analysis of TE expression in distinct cell types

(A) UMAP representation of the highly expressed RMER17A-int family and **(B)** the MMERVK10C-int family and cluster identities are shown in Fig. 4. Embryonic day 18.5 (E18.5) *Trim28*$^{\Delta/\Delta}$ (*n*=2) and control RNA (*n*=2).

Development • Supplementary information

Figure S6

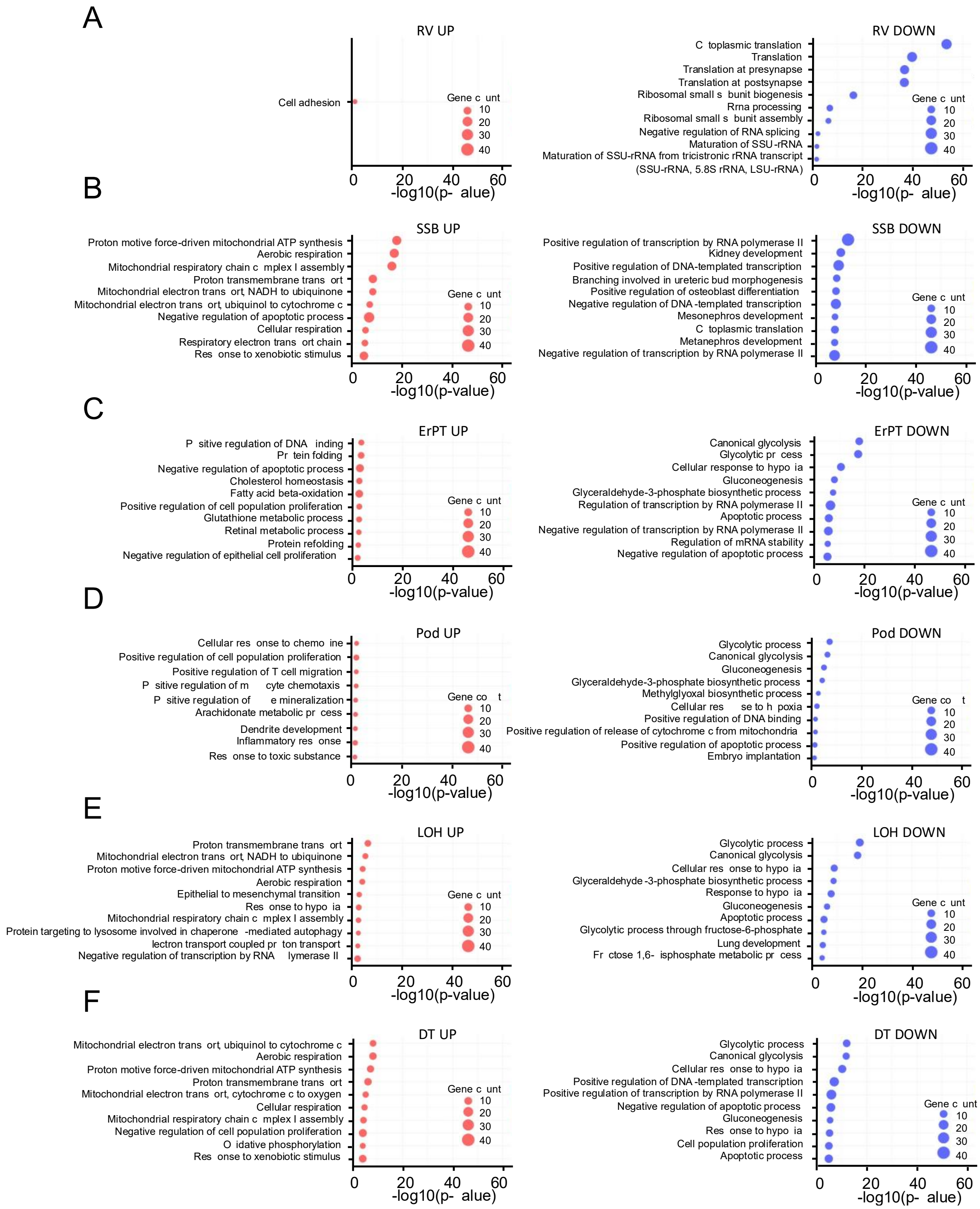

Fig. S6. GO term analysis of kidney structures

GO term analysis of DEGs in **(A)** renal vesicle (RV) **(B)** S-shaped body (SSB) **(C)** early proximal tubule (ErPT) **(D)** podocytes (Pod) **(E)** loop of Henle (LOH) **(F)** distal tubule (DT). Embryonic day 18.5 (E18.5) *Trim28*$^{\Delta/\Delta}$ (*n*=2) and control RNA (*n*=2).

Development • Supplementary information

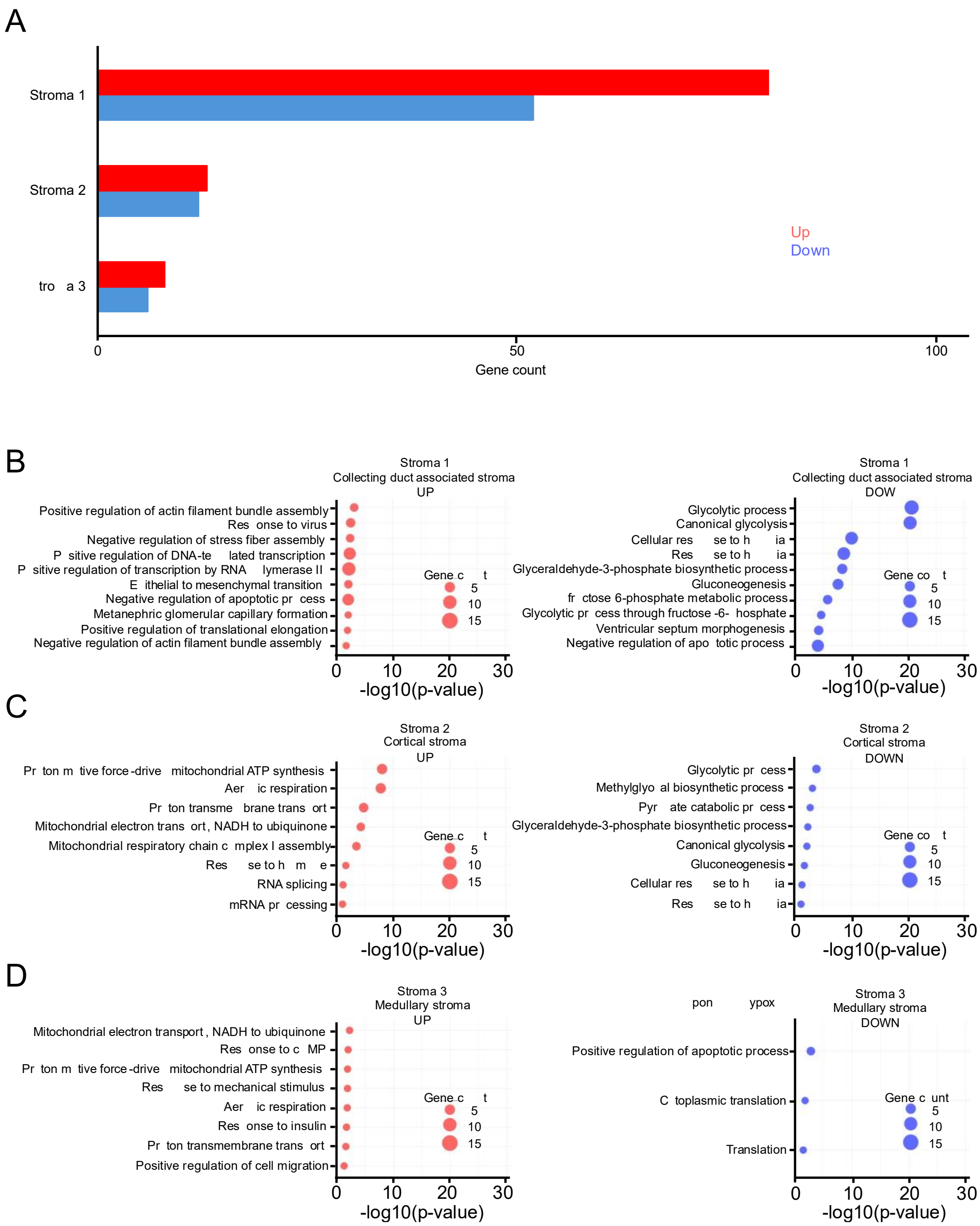

Fig. S7. Differential gene expression and GO term analysis in stroma clusters

(A) Number of differentially expressed genes (DEGs) in stroma cells: Stroma 1 – collecting duct–associated stroma, Stroma 2 – cortical stroma, Stroma 3 – medullary stroma. **(B–D)** Gene Ontology (GO) term analysis of DEGs in **(B)** Stroma 1, **(C)** Stroma 2, and **(D)** Stroma 3. Embryonic day 18.5 (E18.5) *Trim28*$^{\Delta/\Delta}$ (*n*=2) and control RNA (*n*=2).

Development • Supplementary information

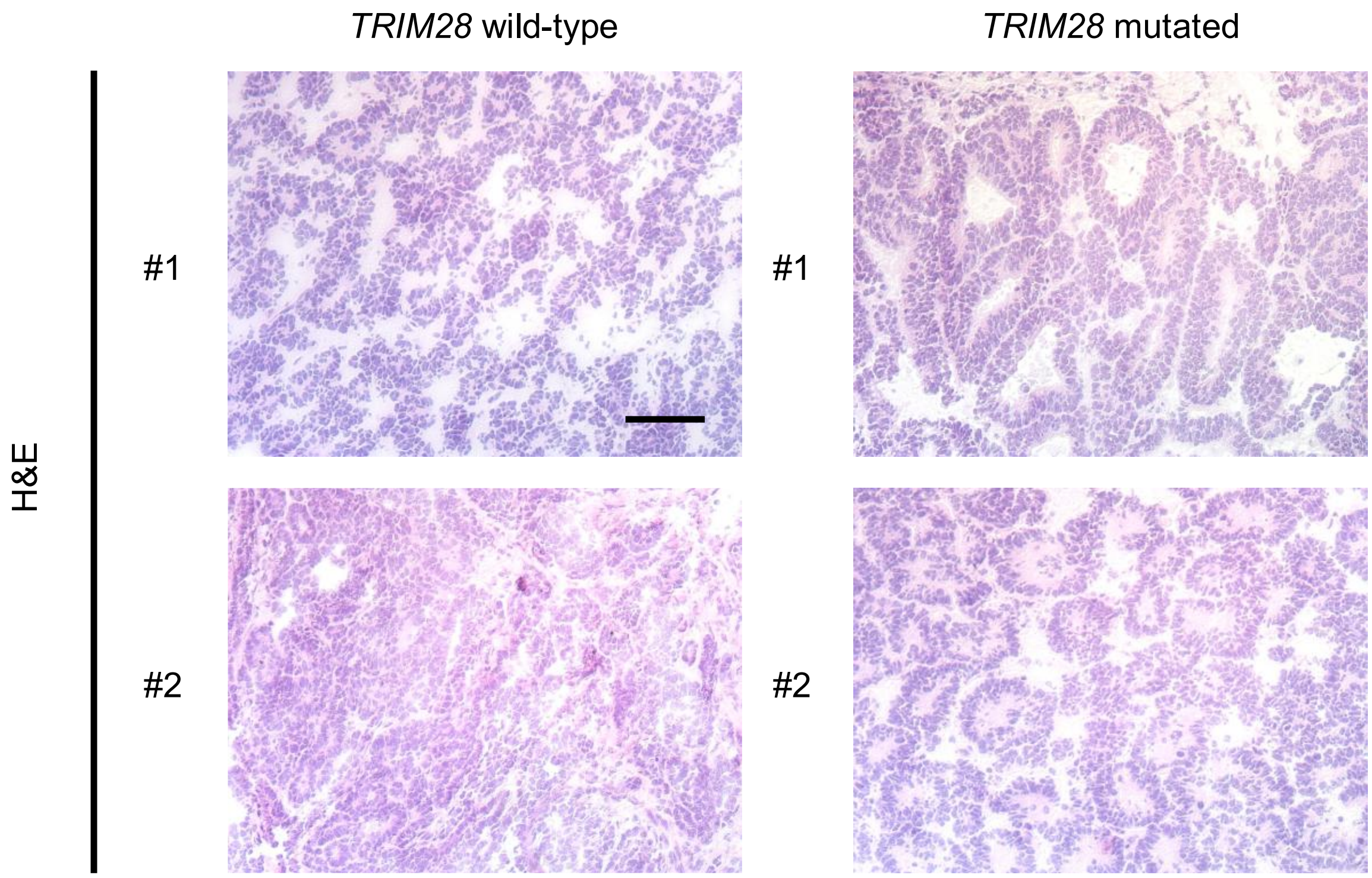

Fig. S8. Histological analysis of *TRIM28* wild-type and *TRIM28* mutated WT human tumor samples

Hematoxylin and eosin (H&E) staining of *TRIM28* wild-type (*n*=2) and *TRIM28* mutant (*n*=2) Wilms tumor (WT) samples. Scale bar represents 100 µm.

Development • Supplementary information

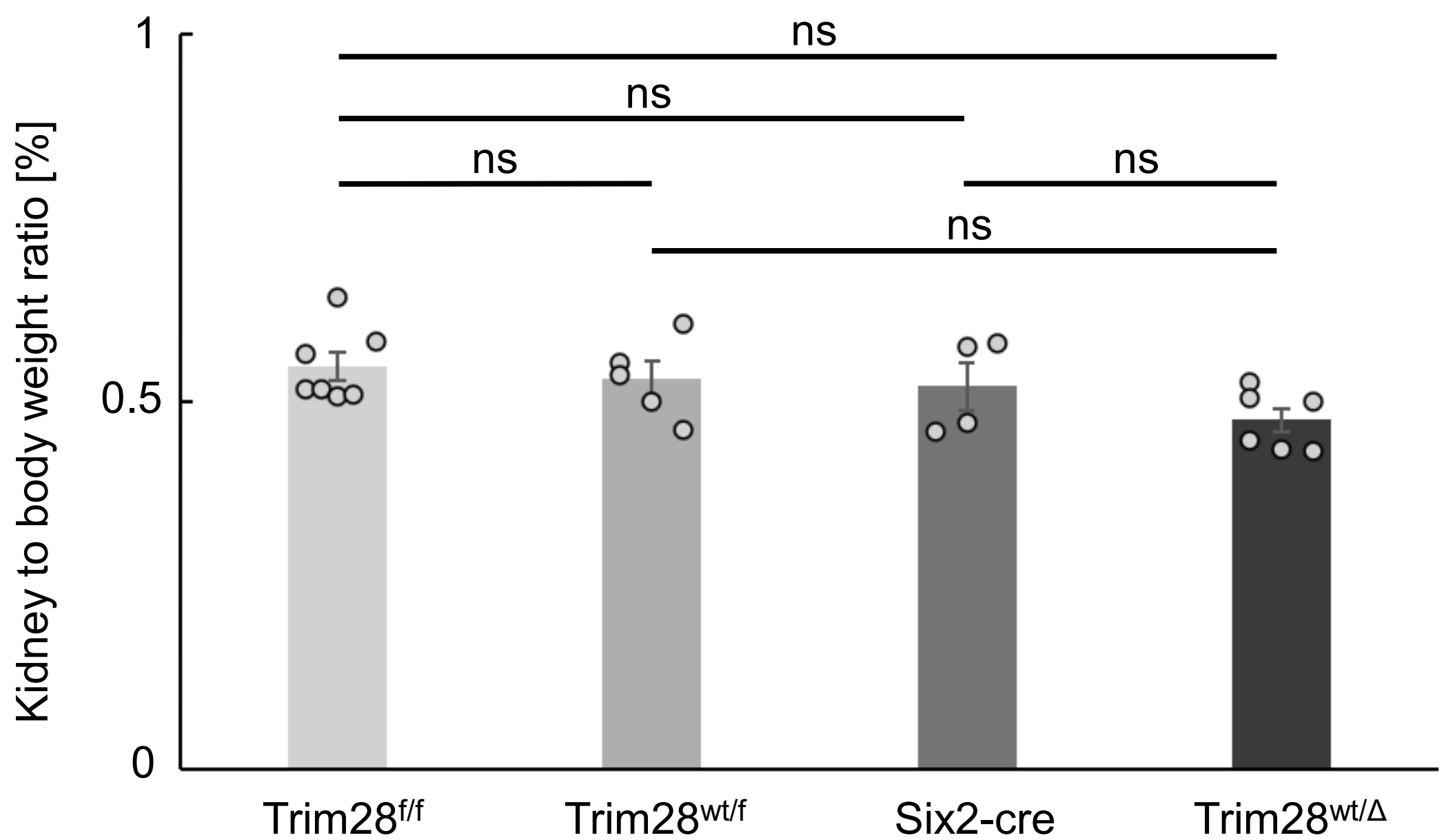

Fig. S9. Comparison of the kidney-to-body weight ratio in newborn (P0) *Trim28*$^{f/f}$, *Trim28*$^{wt/f}$, six2-cre and *Trim28*$^{wt/\Delta}$mice. Animal numbers that were used for the study: *Trim28*$^{f/f}$ (*n*=7), *Trim28*$^{wt/f}$ (*n*=5), Six2-cre (*n*=4) and Trim28$^{wt/\Delta}$ (*n*=6). ns = not significant (two-sided Welch's t-test).

Table S1. Differentially expressed genes between knockout and control samples from bulk RNA sequencing

Available for download at
https://journals.biologists.com/dev/article-lookup/doi/10.1242/dev.205188#supplementary-data

Table S2.Top 100 differentially expressed transposable elements identified between knockout and control samples

Available for download at
https://journals.biologists.com/dev/article-lookup/doi/10.1242/dev.205188#supplementary-data

Development • Supplementary information

Table S3. Deregulated transposable element families

Available for download at

https://journals.biologists.com/dev/article-lookup/doi/10.1242/dev.205188#supplementary-data

Table S4. List of upregulated genes with upregulated transposable elements within a 50 kb distance in knockout samples

Available for download at

https://journals.biologists.com/dev/article-lookup/doi/10.1242/dev.205188#supplementary-data

Table S5. List of downregulated genes with upregulated transposable elements within a 50 kb distance in knockout samples

Available for download at
https://journals.biologists.com/dev/article-lookup/doi/10.1242/dev.205188#supplementary-data

Table S6. List of primers used in this study

Available for download at

https://journals.biologists.com/dev/article-lookup/doi/10.1242/dev.205188#supplementary-data

Supplementary Materials and Methods

Histopathology and immunohistochemistry

H&E and immunohistochemical stainings were conducted as described in the Methods section of the manuscript. The primary antibodies used were rabbit anti-Six1/2 (1:500, Sigma, HPA001893) and rat anti-Ki67 monoclonal antibody (SolA15) (1:50, Invitrogen, Thermo Fisher Scientific).

Six2-positive and Six2/Ki67 double-positive cells (Fig. S3) were determined manually from 3 individuals per genotype. One or two slides were stained from each individual, and two or three fields of a kidney were imaged. A total of 14 and 15 fields were quantified for Trim28$^{f/f}$ and Trim28$^{\Delta/\Delta}$, respectively. Six2-positive cells are organized in distinct structures along the kidney perimeter. Therefore, the cell numbers were expressed as cells per length of perimeter within a field. After the Kolmogorov-Smirnov test revealed that genotype values were normally distributed, an unpaired two-sided t-test with Welch's correction was used to determine the significance of the differences.

Ultrastructural studies using semi-thin kidney sections

For ultrastructural analysis, kidneys from 3 Trim28$^{\Delta/\Delta}$ and 2 Trim28$^{f/f}$ mice were fixed in 4% formalin buffered in 0.1M PBS, pH 7.6. After washing in 0.1 M PBS, pH 7.6, kidneys were dehydrated in an ascending series of alcohols and tissue was embedded in epoxy Araldite resin (Serva Electrophoresis GmbH, Heidelberg, Germany). Semi-thin sections with a thickness of 0.5 µm were cut on a UC6 ultramicrotome (Leica, Wetzlar, Germany), stained with toluidine blue and digitalized using a Zeiss Z1 whole slide scanner (Zeiss, Oberkochen, Germany).

To determine the number of glomerular capillary sections and the area of each sectioned capillary lumen in the glomeruli, all mature glomeruli of both genotypes were evaluated. The lumina of the glomerular capillaries were traced using Zen software (Zeiss), and the areas were measured.

The glomerular tuft areas were also manually determined for premature and mature glomeruli. Following the Kolmogorov-Smirnov test, which revealed that the values in the genotypes were non-normally distributed, the Mann-Whitney test was used to check the significance of the determined differences.

Development • Supplementary information
