# Supplementary material for: The absence of Trim28 in nephron progenitors results in impaired kidney development and function
Source: Development. 2026 Aug 13;153(15):dev205188. doi: 10.1242/dev.205188 (PMC13535099; doi:10.1242/dev.205188)
Supplement: Supplementary file 2 [file develop-153-205188_review_history.pdf]

The absence of *Trim28* in nephron progenitors results in impaired kidney development and function

Balazs Palhazi, Alireza Paikari, Greta Clarén, Silke Appenzeller, Fabian Imdahl, Christoph Daniel, Matthias Becker and Manfred Gessler

DOI: 10.1242/dev.205188

Editor: James M Wells

Review timeline
Original submission: 22 August 2025
Editorial decision: 2 October 2025
First revision received: 24 April 2026
Editorial decision: 13 May 2026
Second revision received: 6 July 2026
Accepted: 12 July 2026

Original submission

First decision letter

MS ID#: dev.205188

MS TITLE: The absence of Trim28 in nephron progenitors results in impaired kidney development and function

AUTHORS: Balazs Palhazi, Alireza Paikari, Silke Appenzeller, Fabian Imdahl, Matthias Dr Becker and Manfred Gessler

Dear Dr Becker,

I have now received all the referees' reports on the above manuscript, and have reached a decision. The referees' comments are appended below, or you can access them online: please go to:

As you will see, the referees express considerable interest in your work, but have some significant criticisms and recommend a substantial revision of your manuscript before we can consider publication. If you are able to revise the manuscript along the lines suggested, which may involve further experiments, I will be happy to receive a revised version of the manuscript. Your revised paper will be re-reviewed by one or more of the original referees, and acceptance of your manuscript will depend on your addressing satisfactorily the reviewers' major concerns. Please also note that Development will normally permit only one round of major revision. If it would be helpful, you are welcome to contact us to discuss your revision in greater detail. Please send us a point-by-point response indicating your plans for addressing the referees' comments, and we will look over this and provide further guidance.

Please attend to all of the reviewers' comments and ensure that you upload both a 'clean' version of your Word file, along with a highlighted version clearly showing where you have made changes in the revised manuscript. Please avoid using 'Tracked changes' in Word files as these are lost in PDF conversion. I should be grateful if you would also provide a point-by-point response detailing how you have dealt with the points raised by the reviewers in the 'Response to Reviewers' box. If you do not agree with any of their criticisms or suggestions please explain clearly why this is so.

© 2026. Published by The Company of Biologists under the terms of the Creative Commons Attribution License (https://creativecommons.org/licenses/by/4.0/).

Reviewer 1

SUMMARY OF THE ADVANCE MADE IN THIS PAPER AND ITS POTENTIAL SIGNIFICANCE TO THE FIELD

This interesting study describes a kidney phenotype of mice with conditional deletion of the transcriptional regulator TRIM28 in nephrogenic progenitor cells. The authors use state-of-the art technology to demonstrate a cell-type-specific dysregulation of retroelement expression with a reduction of NPC counts and an increase in (abnormal) proximal tubular cells in Trim28-deficient kidneys. Differential gene expression analysis suggests reduced translation in Trim28-deficient NPCs, which is also evident in TRIM28-mutant Wilms tumor tissue.

SUGGESTIONS TO AUTHORS

Major comments

1. Kidney mass is significantly reduced in newborn Trim28Δ/Δ mice (Fig. 2D, E), possibly due to diminished NPC counts (Fig. 4A). What is the reason for the NPC reduction in Trim28Δ/Δ kidneys? Cell cycle analysis based on phase-specific marker gene expression suggests reduced proliferation of NPCs in Trim28-deficient kidneys (Fig. 4C). However, this difference between Trim28Δ/Δ and Trim28fl/fl NPCs appears relatively small and may not fully explain the reduced NPC counts. Apoptotic loss of NPCs might be considered as a possible reason for the diminished NPC pool in Trim28Δ/Δ kidneys.

2. Nephrogenic progenitor cells give rise also to glomerular podocytes and parietal epithelial cells of Bowman's capsule. This raises the question of whether Trim28Δ/Δ mice display glomerular lesions as might be expected from their reduced Wt1 expression (Fig. 2D).

3. Fig. 3A: The heat map showing differentially expressed transposable elements in Trim28Δ/Δ mice lacks labeling. Please, add transcript names to the heat map or provide transcripts in a separate table.

4. Information on the number of animals/ kidney specimens (Trim28Δ/Δ controls) examined should be provided for all experiments.

Minor comments

5. Fig. 1D: Labeling of the Y-axis (kidney to body weight ratio) is missing

6. Page 5, lines 10-12: "We saw small and non-significant changes comparing C57/BL6, Six2-cre and Trim28f/f newborns, which may be due to minor differences in genetic background or statistical variations."
Please, describe in a bit more detail what these changes consist of (differences in kidney mass/weight/ structure etc.?).

Reviewer 2

SUMMARY OF THE ADVANCE MADE IN THIS PAPER AND ITS POTENTIAL SIGNIFICANCE TO THE FIELD

In this manuscript, Palhazi et al describe a conditional knockout of Trim28 in the nephron progenitor cells using a Six2-Cre driver. TRIM28 is a commonly mutated Wilms tumors (especially with epithelial histology), and the NPCs are the best candidate for the cell of origin, the rationale for this model is beyond doubt. However, as with other Wilms tumor models, the data is clear but the interpretation is challenging to say the least. The authors describe early postnatal lethality, and focus because of that on P0 and E18.5 samples. They describe morphological changes in the tubules, especially the proximal tubule. The proximal tubule is also the most affected part of the developing kidneys in bulk RNA-seq analysis. And scRNA-seq analysis shows a decrease in NPCs and increase in PTCs. Some of the changes found in the mutant NPCs with respect to translational genes were also found in scRNA-seq data from TRIM28 mutant Wilms tumors. So at a surface several things make sense. However, when digging deeper a lot of unexpected and unexplanable things are found.

© 2026. Published by The Company of Biologists under the terms of the Creative Commons Attribution License (https://creativecommons.org/licenses/by/4.0/).

The mixing of DT and PT cells is dififcult to explain, but could suggest some patterning phenotype. However, patterning is a post-MET process (although the model presented by Lindstrom et al (2018), PMID 29870722) would bring this forward to the moment and order of cells passing through the cap mesenchyme, and could be more in line with a patterning problem caused by a mutation in NPCs). There is an overlap in the transcriptional response with NPCs from TRIM28 mutant tumors, but whereas the mouse model kidneys show decrease in NPCs and increase in PT, whereas in the human tumors there is an increase in NPCs. I assume that this increase in NPCs whereas the TRIM28 tumours are more epithelial will be discussed in the 'in preparation' manuscript, but the difference with the mouse model here is also remarkable. There are other things I noticed in the data as described which I will discuss below.

In short, there are a lot of really interesting observations in this model, that are really hard to interpret, not from a developmental point of view and not from a Wilms tumor point of view. It is also difficult to pinpoint clear additional experiments that in my opinion could realistically be expected in a revision. My main request to the authors is therefore to clarify certain things and to speculate more on others.

SUGGESTIONS TO AUTHORS

The authors mention early postnatal lethality of the model. There isn't any detail abuot this. How early do the mtuants die? Is this within hours (or even quicker) after birth, or are we talking about a few days? Is there variation in this? Linked to this is the cause of death. If mouse pups have no kidney function at all they die within minutes of birth (and the cadavers are often eaten by the mother before the pups can even be found). On the ohter hand, I have seen cases where the kidneys look really disastrous but still the pups survive for a few weeks. Just looking at the morphology of the mutant kidneys as presented I would really be surprised if this was the cause of death. Did the authors look at other sites where Six2 expression overlaps with Wt1 at some point during development? The developing pallate and stomach come to mind.

The down regulation of Wnt4 in the medulla (fig. 2D) is mentioned but not discussed, but it is really striking and convincing, but is unexpected with a knock put in the nephrogenic lineage. Wnt4 is indeed a marker for PTAs, and these are in the nephrogenic lineage, but these seem normal. The Wnt4 in the medulla is stromal lineage, and altnough clearly lost, this is not in the Six2 lineage. Clearly the loss of WNt4 in the medula must be some indirect effect, for instance through disturbed communicaiton bewteen the lineages. Could the authors discuss this? Linked to this is the decrease in stromal cells as suggested in fig 4A and confirmed in 4B. Even if this reduction in cells is not as big as for instance the NPCs, if drastic things happen in these cells, like Wnt4 (or Rarb which seems to go the other way in the medulla), there might be interesting expression changes in these cells. Yet the stroma is not included in the DEG analysis in fig S4. Even if not expected in the Six2-Cre mutants, it might be wotrthwhile to look at this as well.

THe reduction in NPCs and increase in PT cells is striking, but the section title 'a shift from nephron progenitors to proximal tubule cells' might be suggesting more than can be supported right now. At least for me, this sounds like a developmental shift, for instance increased speed of differentiation and increased proliferation of the PT cells. This would be an interesting thing, but I don't think this is the only option. If this is indeed what the autnhors want to claim, I think data of earlier timepoints should be studied (E11.5 onwards) to get an idea of the dynamics of this. ALso parameters like proliferation and apaptosis should be checked (this could all be done on sections). Another interesting question would then also be why only PT cells increase and not other post-MET cell types. If the authors don't intend to make this claim I think the title of the section should be different.

The mutant mice don't get Wilms tumors. THe simplest factor here is they simply die too quickly, but I'm actually wondering if the Six2-Cre is the best model for this. Wilms tumors with TRIM28 loss are epithelial. By hitting Trim28 already in the NPCs the authors might be affecting the NPCs becoming epithelial cells, whereas hitting the gene after the MET and cells are already epithlialized might have a different effect, and might just as well be what happens in the patient. The mentioned alternative of a Cited1-CreERT2 woud with low dose tamoxifen give the option of extending the lifespan of the mutants (but this is in practice a lot more dififcult than it sounds) but won't change this timing. I would argue that a post-MET driuver, for instance Ksp-Cadherin-Cre (aka

© 2026. Published by The Company of Biologists under the terms of the Creative Commons Attribution License (https://creativecommons.org/licenses/by/4.0/).

Cdh16-Cre) might be at least as informative. Could the authors speculate on this? THis is not to say the Six2-Cre data is worthless, in contrast. Not seeing the expected, or hoped for, things in a mouse model can be just as informative as seeing them.

In short, I really like the data presented in this paper even though it is difficult to draw form conclusions from them with our current understanding.

Reviewer 3

SUMMARY OF THE ADVANCE MADE IN THIS PAPER AND ITS POTENTIAL SIGNIFICANCE TO THE FIELD

The study presents a novel and compelling role for TRIM28 in nephron progenitor cell maintenance and differentiation. The link to Wilms tumor biology adds translational relevance. The manuscript makes a meaningful contribution to developmental nephrology and cancer genetics.

SUGGESTIONS TO AUTHORS

1. Some recent studies (2024-2025) that would be valuable additions to the manuscript for contextualizing and strengthening its findings:

A. Whitworth et al. (2024) Wilms tumour resulting from paternal transmission of a TRIM28 pathogenic variant.

B. Wegert et al. (2025) Distinct pathways for genetic and epigenetic predisposition in familial and bilateral Wilms tumor.

C. Li et al. (2024) USC-led study introduces a new and improved way to grow nephron progenitor cells.

D. Araoka et al. (2025) Human iPSC-derived nephron progenitor cells treat acute kidney injury and chronic kidney disease in mouse models.

2. While the RNA-seq and scRNA-seq data are well-analyzed, the sample sizes (N=2 for scRNA-seq) are relatively small. The authors should discuss the limitations this may impose on statistical power and reproducibility.

3. The deregulation of transposable elements (TEs) is intriguing. The authors should consider validating the functional impact of TE activation on nearby gene expression or inflammatory pathways, possibly through reporter assays or CRISPR-based silencing.

4. The absence of tumor formation in Trim28^Δ/Δ^ mice is acknowledged, but the discussion could be expanded to address whether early lethality or lack of secondary mutations may explain this outcome. Future directions using inducible or mosaic models would be valuable.

5. The increase in proximal tubule cells could reflect accelerated differentiation, compensatory proliferation, or selective survival. The authors should explore alternative explanations and consider lineage tracing or proliferation assays to clarify this.

6. Some figures, particularly in the supplementary section, appear pixelated or compressed. Higher-resolution images and consistent formatting would improve readability and data interpretation.

7. The discussion could benefit from deeper integration with known pathways in nephrogenesis (e.g., Wnt, Notch, BMP) and how TRIM28 may intersect with these signaling networks.

8. Given the parallels with TRIM28-mutant Wilms tumors, the authors should elaborate on potential diagnostic or therapeutic implications, such as TE regulation as a biomarker or TRIM28 as a target in pediatric renal malignancies.

9. Although the authors note that Six2-cre alone does not affect kidney size in their background, a brief mention of potential off-target recombination or background effects would be prudent.

First revision

Author response to reviewers' comments

Reviewer 1: SUMMARY OF THE ADVANCE MADE IN THIS PAPER AND ITS POTENTIAL SIGNIFICANCE TO THE FIELD

© 2026. Published by The Company of Biologists under the terms of the Creative Commons Attribution License (https://creativecommons.org/licenses/by/4.0/).

This interesting study describes a kidney phenotype of mice with conditional deletion of the transcriptional regulator TRIM28 in nephrogenic progenitor cells. The authors use state-of-the art technology to demonstrate a cell-type-specific dysregulation of retroelement expression with a reduction of NPC counts and an increase in (abnormal) proximal tubular cells in Trim28-deficient kidneys.

Response: Thank you for this positive statement on our work.

Response to Major comments:

1. Kidney mass is significantly reduced in newborn Trim28∆/∆ mice (Fig. 2D, E), possibly due to diminished NPC counts (Fig. 4A). What is the reason for the NPC reduction in Trim28∆/∆ kidneys? Cell cycle analysis based on phase-specific marker gene expression suggests reduced proliferation of NPCs in Trim28-deficient kidneys (Fig. 4C). However, this difference between Trim28∆/∆ and Trim28fl/fl NPCs appears relatively small and may not fully explain the reduced NPC counts. Apoptotic loss of NPCs might be considered as a possible reason for the diminished NPC pool in Trim28∆/∆ kidneys.

Response: We thank the reviewer for this comment. To support the marker gene expression data shown in Fig. 4B, C we have now performed immunofluorescence staining of E17.5 kidney sections using anti- Six2 and anti-Ki67 antibodies. Six2 served as a marker for NPCs and Ki67 as marker for proliferating cells. Our results show that, in agreement with the marker gene studies, NPC frequencies are clearly reduced in Trim28$^{\Delta/\Delta}$ kidneys. In further agreement with the marker gene analyses, we find only a slight reduction of the relative number of proliferating Trim28$^{\Delta/\Delta}$ NPCs. We show this data in a new supplementary figure (Fig. S3) and describe this figure in the results section on page 9, lines 6-9 as follows: "This finding is supported by immunofluorescence analyses of Six2-positive NPCs in E17.5 kidneys (Fig S.3). Interestingly, the reduced frequency of NPCs is accompanied only by a slight decrease in proliferation (Fig. S3 and Fig. 4B)."

We agree with the reviewer that apoptotic loss would be a plausible explanation for the reduced NPC frequency upon Trim28 loss. Alternatively, a combination of accelerated differentiation and reduced stem cell maintenance (reduced self-renewal capacity) would be an explanation for the observed reduced NPC numbers upon Trim28 deletion (see also our comment to Point 5 of reviewer 3). To precisely identify the contribution of differentiation, stem cell self-renewal and apoptosis one would need to employ lineage tracing experiments in addition to the analysis of cell death in the NPC compartment. Since this would require extensive breeding of mice to harvest enough embryos with correct transgene expression, we feel that this set of analyses is beyond the scope of the current manuscript We have now included a statement referring to this point in the "Limitations of the study" section at page 16 lines 29-31 and page17 lines 1-5 of the manuscript and now write: "Additionally, while our data show that Trim28$^{\Delta/\Delta}$ NPC frequencies decreased and PT cell frequencies increased, we currently do not have an explanation for the underlying mechanisms that led to these results. Although we cannot exclude apoptotic loss of Trim28$^{\Delta/\Delta}$ NPCs, a plausible explanation for the different NPC and PT cell numbers is a combination of accelerated differentiation and reduced stem cell maintenance. Future studies clarifying this question will require genetic lineage tracing, the gold standard for such tests.

2. Nephrogenic progenitor cells give rise also to glomerular podocytes and parietal epithelial cells of Bowman's capsule. This raises the question of whether Trim28∆/∆ mice display glomerular lesions as might be expected from their reduced Wt1 expression (Fig. 2D).

Response: We thank the reviewer for raising this interesting point. To assess glomerular integrity, we have analysed semi-thin sections of Trim28$^{\Delta/\Delta}$ and Trim28$^{f/f}$ P0 kidneys. Interestingly, we find that glomeruli did not show lesions upon *Trim28* deletion. However, we did notice that the number of capillaries per glomerulus was significantly reduced. This reduction was paralleled by increased capillary cross-sectional lumen area. As of now we do not know what causes these changes to the glomerular capillaries. One interesting lead could be

© 2026. Published by The Company of Biologists under the terms of the Creative Commons Attribution License (https://creativecommons.org/licenses/by/4.0/).

the reduced podocyte frequency in Trim28$^{\Delta/\Delta}$ kidneys (Fig 4B in our revised manuscript), as podocytes are involved in the endothelial cell migration, survival, and differentiation in the glomerular tuft (Vaughan MR and Quaggin SE 2008). We show this data in a new supplementary Figure (Fig. S4) and describe this figure in the results section on page 9, lines 9-15. as follows: "In addition to the reduced NPC numbers, more differentiated cell types, such as podocytes, also exhibited a decreased frequency following *Trim28* deletion. Notably, the reduced podocyte frequency was accompanied by a significant reduction in the number of capillaries per glomerulus and a pronounced increase in capillary cross-sectional lumen area, as well as a slight reduction in glomerular size. Nevertheless, *Trim28* deletion had no effect on the overall structure and integrity of the glomerulus (Fig. S4A-D)."

We also discuss this finding in the revised discussion section and now write on page 13 lines 1-5: "Although the reduced number of glomerular capillaries and the pronounced increase in capillary cross-sectional lumen area are most likely not lethal by themselves, they do add complexity to the phenotype of Trim28$^{\Delta/\Delta}$ kidneys. Consequently, the precise cause of death of the animals is not fully understood".

3. Fig. 3A: The heat map showing differentially expressed transposable elements in Trim28Δ/Δ mice lacks labeling. Please, add transcript names to the heat map or provide transcripts in a separate table.

Response: We now provide the transcript names in a new supplementary table (Table S2) and refer to this table in the revised version of the manuscript on page 7, line 29

4. Information on the number of animals/ kidney specimens (Trim28Δ/Δ, controls) examined should be provided for all experiments.

Response: We now provide information on the number of animals/ kidney specimens (Trim28$^{\Delta/\Delta}$, controls) in the legends to figures 1- 4.

<u>Response to minor comments:</u>

5. Fig. 1D: Labeling of the Y-axis (kidney to body weight ratio) is missing

Response: We have included the Labelling of the Y- axis and now also show the individual data points in the graph in Figure 1D. We also included labelling for the Y-axis in Fig. 4B + C and Fig. 6B of the revised manuscript.

6. Page 5, lines 10-12: "We saw small and non-significant changes comparing C57/BL6, Six2-cre and Trim28f/f newborns, which may be due to minor differences in genetic background or statistical variations."
Please, describe in a bit more detail what these changes consist of (differences in kidney mass/ weight/ structure etc.?).

Response: The Six2Cre mice were bred by crossing them with wildtype C57Bl/6 mice for heterozygous propagation of the Six2Cre transgene, whereas the Trim28f/f mice were maintained by constant interbreeding. The Trim28f/f mice were backcrossed to the wildtype C57Bl/6 background every two to three years. Figure R1A shows the body weights of C57Bl/6, Six2Cre, Trim28f/f and Trim28$^{\Delta/\Delta}$ newborns. The data show that C57Bl/6 and Six2Cre newborns had very similar body weights. Compared to these mice, the Trim28f/f and Trim28Δ/Δ mice reached slightly reduced body weights. We hypothesise that this is due to subtle genetic differences caused by the different breeding strategies. The kidney weights of the newborns (Fig. R1B) are generally proportional to the body weights. Only the Trim28$^{\Delta/\Delta}$ animals show disproportionally smaller kidneys as compared to the kidneys of all other mouse lines as shown by the significantly lower kidney to body weight ratio (Fig. 1D in our revised manuscript). As our paper focuses on the differences brought about by Trim28 deletion, we feel that including information on the body and kidney weights of the individual mouse lines distracts from our point, and we therefore decided to leave this data out of the manuscript. However, it can be included if the reviewer deems it necessary.

© 2026. Published by The Company of Biologists under the terms of the Creative Commons Attribution License (https://creativecommons.org/licenses/by/4.0/).

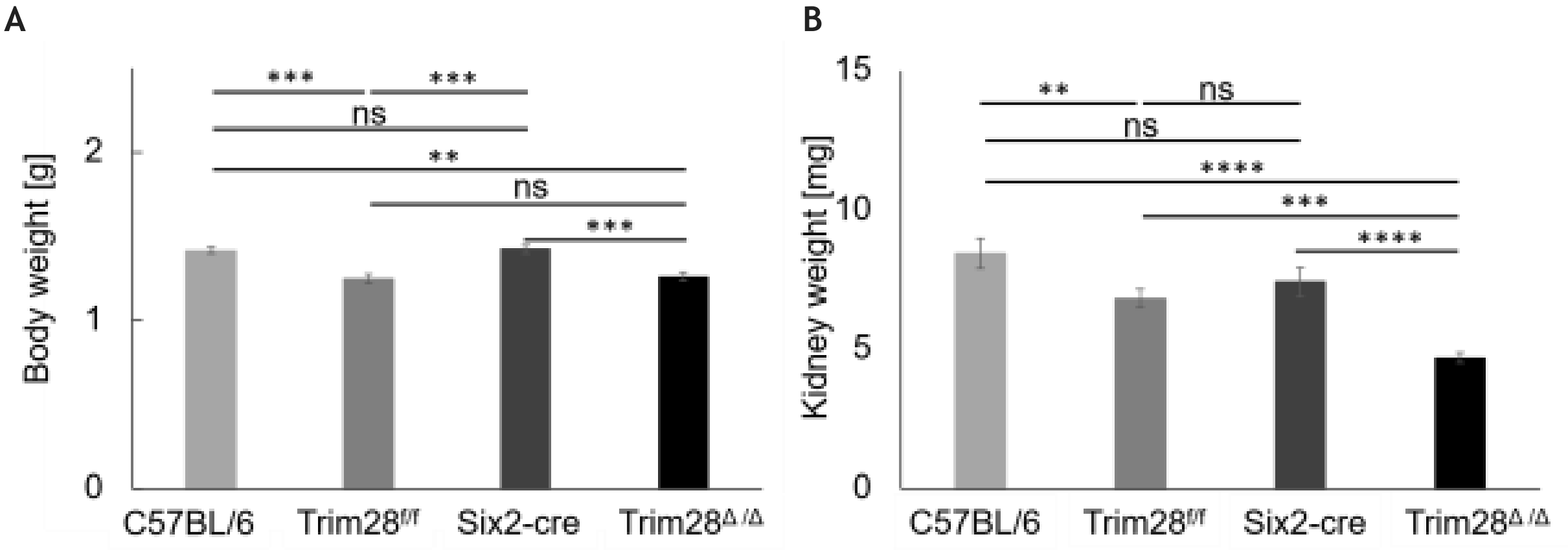

Figure R1. Body and kidney weight of C57Bl/6, Six2Cre, Trim28f/f and Trim28Δ/Δ mice Comparison of body (A) and kidney (B) weights in wild-type C57BL/6, Trim28$^{f/f}$, Six2-Cre, and Trim28$^{\Delta/\Delta}$ mice. Trim28$^{\Delta/\Delta}$ mice exhibit significantly lower kidney weights compared to controls. Bars represent mean ± s.e.m.. Sample sizes: C57BL/6 (n = 4), Trim28$^{f/f}$ (n = 7), Six2-Cre (n = 4), and Trim28$^{\Delta/\Delta}$ (n = 9).

Reviewer 2: SUMMARY OF THE ADVANCE MADE IN THIS PAPER AND ITS POTENTIAL SIGNIFICANCE TO THE FIELD

In this manuscript, Palhazi et al describe a conditional knockout of Trim28 in the nephron progenitor cells using a Six2-Cre driver. TRIM28 is a commonly mutated Wilms tumors (especially with epithelial histology), and the NPCs are the best candidate for the cell of origin, the rationale for this model is beyond doubt. However, as with other Wilms tumor models, the data is clear but the interpretation is challenging to say the least. The authors describe early postnatal lethality, and focus because of that on P0 and E18.5 samples. They describe morphological changes in the tubules, especially the proximal tubule. The proximal tubule is also the most affected part of the developing kidneys in bulk RNA-seq analysis. And scRNA-seq analysis shows a decrease in NPCs and increase in PTCs. Some of the changes found in the mutant NPCs with respect to translational genes were also found in scRNA-seq data from TRIM28 mutant Wilms tumors. So at a surface several things make sense. However, when digging deeper a lot of unexpected and unexplanable things are found. The mixing of DT and PT cells is dififcult to explain, but could suggest some patterning phenotype. However, patterning is a post-MET process (although the model presented by Lindstrom et al (2018), PMID 29870722) would bring this forward to the moment and order of cells passing through the cap mesenchyme, and could be more in line with a patterning problem caused by a mutation in NPCs).

There is an overlap in the transcriptional response with NPCs from TRIM28 mutant tumors, but whereas the mouse model kidneys show decrease in NPCs and increase in PT, whereas in the human tumors there is an increase in NPCs. I assume that this increase in NPCs whereas the TRIM28 tumours are more epithelial will be discussed in the 'in preparation' manuscript, but the difference with the mouse model here is also remarkable. There are other things I noticed in the data as described which I will discuss below.

Response: Thank you very much for the thoughtful comments on our work and its meaning in the broader context of kidney development and tumor formation. Our data do not indicate mixing of the DT and PT compartment. The different cell types in the kidney were identified based on a set of expressed marker genes that were previously reported in the literature (Combes et al 2019) based on the individual marker genes the cell types were unambiguously assigned for the Trim28$^{f/f}$ and the Trim28$^{\Delta/\Delta}$ kidneys. The higher number of cells with NPC-like expression patterns in human WT may underlie tumor formation as opposed to our mouse model, where the shift towards tubular epithelial differentiation is seen, but the exhaustion of NPCs may preclude tumor formation. To address this relevant point we are now writing in the discussion section on page 16 lines 20-23 "…It may also help to unravel the puzzling

© 2026. Published by The Company of Biologists under the terms of the Creative Commons Attribution License (https://creativecommons.org/licenses/by/4.0/).

observation that NPCs are abundant in human TRIM28-mutant WT but depleted in our mouse model. This in turn suggests that accelerated differentiation and stem cell depletion in the mouse may be decisive factors."

In short, there are a lot of really interesting observations in this model, that are really hard to interpret, not from a developmental point of view and not from a Wilms tumor point of view. It is also difficult to pinpoint clear additional experiments that in my opinion could realistically be expected in a revision. My main request to the authors is therefore to clarify certain things and to speculate more on others.

Response: We thank the reviewer for this positive comment on our model system. Please find our detailed response to the reviewers' comments below.

Resonse to suggestions to authors:

The authors mention early postnatal lethality of the model. There isn't any detail abuot this. How early do the mtuants die? Is this within hours (or even quicker) after birth, or are we talking about a few days? Is there variation in this? Linked to this is the cause of death. If mouse pups have no kidney function at all they die within minutes of birth (and the cadavers are often eaten by the mother before the pups can even be found). On the ohter hand, I have seen cases where the kidneys look really disastrous but still the pups survive for a few weeks. Just looking at the morphology of the mutant kidneys as presented I would really be surprised if this was the cause of death. Did the authors look at other sites where Six2 expression overlaps with Wt1 at some point during development? The developing pallate and stomach come to mind.

Response: We thank the reviewer for raising this important point. The newborns died within hours after birth. We agree with the reviewer that the changes observed in the Trim28$^{\Delta/\Delta}$ kidneys do not appear to be detrimental enough to cause early lethality at first sight. In their initial description of the Six2Cre deleter-mouse line Kobayashi and colleagues (Kobayashi et al., 2008) describe that the transgene besides its expression in the metanephric mesenchyme is also expressed in the developing head, ear and limbs. We assume that the reviewer ment to ask for potential overlapping expression of Six2-Cre transgene with Trim28 not Wt1.

To address this comment, we are now writing on page 5, lines, 3-18 in the results section of our manuscript:

"The mice died within hours of being born. Their overall condition did not point to a possible cause of death, as they all displayed visible milk spots, indicating that they were well-fed. In addition to the kidneys, other organs could potentially be affected in Trim28Δ/Δ animals. Six2 expression is not limited to the developing kidney, but is also detected in multiple embryonic tissues, including the craniofacial mesenchyme, branchial arches, foregut and midgut regions, genital eminence, somites and limb mesenchyme (Oliver et al., 1995; Kobayashi et al., 2008). As Trim28 is expressed at a significant level during embryogenesis and is essential for this process, it is plausible that Six2-cre and Trim28 are co-expressed in some of these cell populations (Messerschmidt et al., 2012). However, no further behavioral or developmental abnormalities were observed in Trim28Δ/Δ newborns compared to their Trim28f/f control littermates. Consequently, we hypothesize that any potential effects in these tissues are likely to be subtle and unlikely to compromise viability. Based on this, we conclude that kidney failure is the most likely cause of death in Trim28$^{\Delta/\Delta}$ animals."

The down regulation of Wnt4 in the medulla (fig. 2D) is mentioned but not discussed, but it is really striking and convincing, but is unexpected with a knock put in the nephrogenic lineage. Wnt4 is indeed a marker for PTAs, and these are in the nephrogenic lineage, but these seem normal. The Wnt4 in the medulla is stromal lineage, and altnough clearly lost, this is not in the Six2 lineage. Clearly the loss of WNt4 in the medula must be some indirect effect, for instance through disturbed communicaiton bewteen the lineages. Could the authors discuss this? Linked to this is the decrease in stromal cells as suggested in fig 4A and confirmed in 4B. Even if this reduction in cells is not as big as for instance the NPCs, if drastic things happen in these cells, like Wnt4 (or Rarb which seems to go the other way in the medulla), there might be interesting expression changes in these cells.

© 2026. Published by The Company of Biologists under the terms of the Creative Commons Attribution License (https://creativecommons.org/licenses/by/4.0/).

Yet the stroma is not included in the DEG analysis in fig S4. Even if not expected in the Six2-Cre mutants, it might be wotrthwhile to look at this as well.

Response: We agree with the reviewer that the effect on stromal cell types in Trim28$^{\Delta/\Delta}$ kidneys is very interesting. As requested by the reviewer we now included the numbers and the GO term analyses of the differentially expressed genes in a new supplementary figure (Fig. S7). We describe this new supplementary figure in the results section on page 10, lines 13-19 as follows: "Of note the stromal compartment, which is not derived from Six2-positive metanephric mesenchyme, also exhibited differential gene expression in Trim28$^{\Delta/\Delta}$ kidneys compared to the Trim28f/f controls (Fig. S7A-D). This difference was particularly evident in the stromal subpopulation 1, which corresponds to the stroma surrounding the collecting duct. Among the 132 differentially expressed genes we again found genes associated with glycolytic processes and response to hypoxia to be downregulated upon Trim28 loss (Fig. S7A,B)."

We also discuss this point in the discussion section on page 14, lines 3-7 and now write: "It is also worth noting that we found expression changes in the stromal compartment that point to glycolytic and hypoxia-associated processes which further supports the idea that the genetic change in the tubular compartment has an influence on stromal cell populations. "

As suggested by the reviewer we also included a section on the reduced Wnt4 expression in the medulla in the discussion section of page 13, lines 11-17 and now write: "The extent to which the shift towards the PT compartment could affect other cell populations in the kidney is unknown. In this context, it is interesting to note that we found *Wnt4* expression to be strongly reduced in the medulla upon Trim28 deletion. As *Wnt4* is expressed by medullary stromal cells that do not originate from the Six2-positive NPC compartment, an indirect effect emanating from the tubular compartment may lead to the loss of *Wnt4* expression in the medulla. "

The reduction in NPCs and increase in PT cells is striking, but the section title 'a shift from nephron progenitors to proximal tubule cells' might be suggesting more than can be supported right now. At least for me, this sounds like a developmental shift, for instance increased speed of differentiation and increased proliferation of the PT cells. This would be an interesting thing, but I don't think this is the only option. If this is indeed what the autnhors want to claim, I think data of earlier timepoints should be studied (E11.5 onwards) to get an idea of the dynamics of this. ALso parameters like proliferation and apaptosis should be checked (this could all be done on sections). Another interesting question would then also be why only PT cells increase and not other post-MET cell types. If the authors don't intend to make this claim I think the title of the section should be different.

Response: We agree with the reviewer that the title of this section suggests a developmental process We probably would need lineage tracing or fate mapping to fully support this, which is beyond the scope of this manuscript. We have therefore rephrased the title, writing on page 8, line 21-22: "Fewer nephron progenitor cells and more proximal tubule cells in developing Trim28$^{\Delta/\Delta}$ kidneys."

The mutant mice don't get Wilms tumors. THe simplest factor here is they simply die too quickly, but I'm actually wondering if the Six2-Cre is the best model for this. Wilms tumors with TRIM28 loss are epithelial. By hitting Trim28 already in the NPCs the authors might be affecting the NPCs becoming epithelial cells, whereas hitting the gene after the MET and cells are already epithlialized might have a different effect, and might just as well be what happens in the patient. The mentioned alternative of a Cited1-CreERT2 woud with low dose tamoxifen give the option of extending the lifespan of the mutants (but this is in practice a lot more dififcult than it sounds) but won't change this timing. I would argue that a post-MET driuver, for instance Ksp-Cadherin-Cre (aka Cdh16-Cre) might be at least as informative. Could the authors speculate on this? THis is not to say the Six2-Cre data is worthless, in contrast. Not seeing the expected, or hoped for, things in a mouse model can be just as informative as seeing them.

Response: Thank you for bringing this interesting point up. As suggested, we now included a paragraph on the potential use of the Chd16-Cre deleter strain in the discussion and write on page 16, lines 5-16: "…Such a system can induce mosaic inactivation of *Trim28* within the

© 2026. Published by The Company of Biologists under the terms of the Creative Commons Attribution License (https://creativecommons.org/licenses/by/4.0/).

nephrogenic progenitor population and reduce the detrimental effect on kidney development to allow for longer survival of mutant clones and tumor formation to occur. The aforementioned strategies target the fate committed mesenchymal progenitor cell compartment, which gives rise to the tubular epithelial cell types of the nephron through mesenchymal-to-epithelial transition (MET). As it is not known whether the tumor-initiating cell in TRIM28 mutant Wilms' tumors derives from a progenitor cell type that arises before or after MET, it may also be interesting to target the developing nephron at a post-MET stage. In this regard, the Ksp1.3/Cre (Chd16-Cre) system (Shao et al., 2002) could be instrumental, as it enables the specific deletion of target genes in tubular epithelial cells in the developing kidney"

In short, I really like the data presented in this paper even though it is difficult to draw form conclusions from them with our current understanding.

Response: We thank the reviewer for this positive statement on our work.

Reviewer 3: SUMMARY OF THE ADVANCE MADE IN THIS PAPER AND ITS POTENTIAL SIGNIFICANCE TO THE FIELD

The study presents a novel and compelling role for TRIM28 in nephron progenitor cell maintenance and differentiation. The link to Wilms tumor biology adds translational relevance. The manuscript makes a meaningful contribution to developmental nephrology and cancer genetics.
Response: Thank you for this statement on the overall contribution of our work.

Response to suggestions to authors:

1. Some recent studies (2024-2025) that would be valuable additions to the manuscript for contextualizing and strengthening its findings:
A. Whitworth et al. (2024) Wilms tumour resulting from paternal transmission of a TRIM28 pathogenic variant.
B. Wegert et al. (2025) Distinct pathways for genetic and epigenetic predisposition in familial and bilateral Wilms tumor.
C. Li et al. (2024) USC-led study introduces a new and improved way to grow nephron progenitor cells.
D. Araoka et al. (2025) Human iPSC-derived nephron progenitor cells treat acute kidney injury and chronic kidney disease in mouse models.

Response: We thank the reviewer for drawing our attention to these important recent publications. We have carefully reviewed all four papers and have integrated relevant citations into both the Introduction and Discussion sections of the revised manuscript.

Specifically, the studies by Huang et al. (2024, Cell Stem Cell) and Araoka et al. (2025, Science Translational Medicine) have been incorporated to contextualize recent advances in nephron progenitor cell expansion and their translational relevance in the discussion section on page 16, lines 17 and 18. The clinical studies by Wegert et al. (2025, Genome Medicine) and Whitworth et al. (2024, European Journal of Human Genetics) are now cited in a new paragraph in the Introduction on page 4, lines 3-11 were we are now writing: "In the largest cohort of bilateral and familial Wilms tumors to date (129 patients), TRIM28 was confirmed as the second most frequent predisposition gene, with affected tumors showing biallelic inactivation via 19q LOH as essentially the sole oncogenic alteration and a uniform epithelial histology (Wegert et al., 2025). Although Wilms tumor due to germline TRIM28 variants has been predominantly associated with maternal transmission, Whitworth et al. recently documented the first case arising from paternal inheritance, demonstrating that paternal carriers should also be included in genetic counseling (Whitworth et al., 2024)."

We believe these additions strengthen the manuscript and further substantiate the clinical significance of TRIM28 as a Wilms tumor predisposition gene.

© 2026. Published by The Company of Biologists under the terms of the Creative Commons Attribution License (https://creativecommons.org/licenses/by/4.0/).

2. While the RNA-seq and scRNA-seq data are well-analyzed, the sample sizes (N=2 for scRNA-seq) are relatively small. The authors should discuss the limitations this may impose on statistical power and reproducibility.

Response: We agree that a larger sample size would improve the statistical power and reproducibility of the study. Further analyses will therefore be needed to corroborate our findings. However, the independent analyses we performed during the preparation of this manuscript and the subsequent revision process fully support the findings obtained from the single-cell analyses. In line with our single-cell data, the results of our global gene expression analyses (Fig. 2 of the revised manuscript) highlight that the PT segment is strongly affected by Trim28 loss. Furthermore, as demonstrated in our new Figure S3 in the revised manuscript and consistent with our single-cell data, we observe a significant reduction in NPC frequency in E17.5 mice via quantitative immunofluorescence analysis of Six2-positive cells. Consistent with our single-cell data, we observe only a mild reduction in NPC frequency in cycle, as determined by quantifying the Six2/Ki67 double-positive cell fraction. Finally, in single-cell analyses performed with both embryonic mouse kidneys and human tumour tissue, we found a reduction in ribosomal genes in both Trim28-deficient mouse kidney NPCs and Trim28-negative Wilms tumour NPCs compared to their respective controls. We also observed considerable overlap in the genes affected by *Trim28* loss (Fig. 6C, D). These findings suggest that, despite the small sample sizes used, our analyses reveal TRIM28-specific functions that were previously unknown. We are also now writing in the Study limitations section on page 16 lines 26-29: "One limitation of this study is that only two biological replicates were used per genotype in the single-cell RNA sequencing analyses. Future studies would benefit from including a higher number of biological replicates to increase statistical power."

3. The deregulation of transposable elements (TEs) is intriguing. The authors should consider validating the functional impact of TE activation on nearby gene expression or inflammatory pathways, possibly through reporter assays or CRISPR-based silencing.

Response: With almost 400 members in both human and mouse, KRAB-containing zinc finger proteins (KRAB-ZFPs) constitute the largest family of transcriptional regulators in higher vertebrates (Emerson and Thomas, 2009). These genes are expressed in a cell type or tissue type specific manner during development (Zhang et al., 2022). In our understanding the prerequisite to address the reviewer's comment would be to identify cell lines that mimic the situation in differentiating kidney cells. This would include expression of kidney cell-type-specific KRAB-ZFPs which mediate *Trim28* repression at specific TE loci. Additionally, the downstream silencing mechanisms that maintain repression of TEs would have to be inactive in these cells. Furthermore, due to the repetitive nature and the high sequence homology within TE families the targeted elimination of TE sequences via CRISPR/Cas9-directed genome editing is highly complex. The analyses that would have to be conducted to control for possible off target effects would encompass e.g. whole genome sequencing. Given these complexities of stage-specific silencing and the targeted deletion of genomic TE sequences, we feel that these analyses are beyond the scope of the current study.

4. The absence of tumor formation in Trim28^Δ/Δ^ mice is acknowledged, but the discussion could be expanded to address whether early lethality or lack of secondary mutations may explain this outcome. Future directions using inducible or mosaic models would be valuable.

Response: Thank you for these suggestions. We have already mentioned the possibility that tumor formation does not occur in our model because of early lethality in our original version of the manuscript. On page 15, lines 21-23 we write: "While this interesting commonality was observed in mouse and tumor NPCs, no tumor formation was observed in the Six2-cre transgenic animals, most likely due to early lethality". We agree with the reviewer that lack of secondary mutations may be an alternative explanation for non-existing tumor formation in animal models. TRIM28 mutant Wilms tumors are, however, genomically quite stable and, in contrast to other Wilms tumor entities lack shared secondary driver mutations. These findings are reported by Wegert et al 2025. We address this point in the Introduction section and now write on page 4, lines 3-7:" In the largest cohort of bilateral and familial Wilms tumors to date (129 patients), TRIM28 was confirmed as the second most frequent predisposition gene, with affected tumors showing biallelic inactivation via 19q LOH as essentially the sole oncogenic

© 2026. Published by The Company of Biologists under the terms of the Creative Commons Attribution License (https://creativecommons.org/licenses/by/4.0/).

alteration and a uniform epithelial histology (Wegert et al., 2025)". With regard to additional mouse models we refer to the reply to reviewer 2 (Cdh16-Cre mice).

5. The increase in proximal tubule cells could reflect accelerated differentiation, compensatory proliferation, or selective survival. The authors should explore alternative explanations and consider lineage tracing or proliferation assays to clarify this.

Response: This is indeed an important and quite relevant question. As we have shown in Figure 4B of our manuscript and now also show in the new in Fig. S3 of the revised manuscript, there is a reduction in NPCs that is not explained by reduced proliferation. One explanation for the underlying mechanism could be found in a combination of accelerated differentiation and reduced stem cell maintenance that leads to a depletion of the NPC compartment. The reduced proliferation of podocytes or DT cells with slightly elevated PT proliferation rate may not fully explain the differential abundance. Thus, shifting of cell fates may contribute. Genetic lineage tracing as the gold standard for such tests would certainly require large numbers of mice for interbreeding of appropriate transgenes and subsequent analysis that is beyond the scope of this manuscript, however. We have now included a statement in the "Study limitation" section" addressing this point. Please see comment to Reviewer 1. Point 1.

6. Some figures, particularly in the supplementary section, appear pixelated or compressed. Higher-resolution images and consistent formatting would improve readability and data interpretation.

We have now provided the supplementary information in high image resolution.

7. The discussion could benefit from deeper integration with known pathways in nephrogenesis (e.g., Wnt, Notch, BMP) and how TRIM28 may intersect with these signaling networks.

We thank the reviewer for this suggestion. We have included a new paragraph in the discussion section on the possible interaction of Trim28 with key developmental signaling pathways and now write on page 12, lines 11-22: "Reduced NPC frequencies have previously been observed in animals with targeted mutations of factors that are involved in the balancing of NPC self-renewal and differentiation. These factors include members of key signaling pathways such as BMP-, FGF-, and WNT- pathways (*Tomita, M. et al., 2013, Barak, H. et al., 2012, Carroll, T. J., et al., 2005, Karner, C. M. et al., 2011)*. Whereas *Bmp7*-deficient mice show a moderate loss of NPCs that can no longer differentiate, mutations in *Fgf9* and *Fgf20* are associated with a dose- and time- dependent loss of NPCs. A more severe phenotype has been described in mice with targeted Wnt9b deletion, which exhibit disturbed cell cycling and nephron induction, as well as a rapid loss of NPCs. The extent to which Trim28 interacts with these pathways is an interesting question that should be the subject of future investigations".

8. Given the parallels with TRIM28-mutant Wilms tumors, the authors should elaborate on potential diagnostic or therapeutic implications, such as TE regulation as a biomarker or TRIM28 as a target in pediatric renal malignancies.

Although the translation of basic research into clinical applications is highly desirable, unfortunately, the current work cannot contribute much to this effort. Since *TRIM28*-mutant WTs suffer a loss of the protein, the gene or protein itself would not appear to be a suitable therapeutic target. The failure to repress TEs in corresponding tumors seems quite unique to WT, but its diagnostic use would have to rely on liquid biopsy testing of circulating RNA, which is poorly established as yet. In addition, less demanding DNA testing of liquid biopsies to detect *TRIM28* mutations is far more efficient. As there are around 50 potential genetic drivers of WT formation, many of which can be detected in liquid biopsy DNA, such tests will more cost-effective and comprehensive.

9. Although the authors note that Six2-cre alone does not affect kidney size in their background, a brief mention of potential off-target recombination or background effects would be prudent.

© 2026. Published by The Company of Biologists under the terms of the Creative Commons Attribution License (https://creativecommons.org/licenses/by/4.0/).

We thank the reviewer for this comment, which is in agreement with a comment by reviewer 2. As outlined above, we now address this point on page 5, lines, 3-18 in the results section of our manuscript.

References

Emerson, R.O. and Thomas, J.H. (2009) 'Adaptive evolution in zinc finger transcription factors' *PLOS Genetics* **(5)** 1: e1000325

Zhang, Y., He, F., Zhang, Y. et al. (2022) 'Exploration of the regulatory relationship between KRAB-Zfp clusters and their target transposable elements via a gene editing strategy at the cluster specific linker-associated sequences by CRISPR-Cas9' *Mobile DNA* **13** (25)

Kobayashi, A., Valerius, M. T., et al. (2008) 'Six2 defines and regulates a multipotent self-renewing nephron progenitor population throughout mammalian kidney development', *Cell Stem Cell* **3**(2): 169-81.

Vaughan MR, Quaggin SE.' How do mesangial and endothelial cells form the glomerular tuft?' (2008) *Journal of the American Society of Nephrology* Jan;**19**(1):24-33

Second decision letter

MS ID#: dev.205188R1

MS TITLE: The absence of Trim28 in nephron progenitors results in impaired kidney development and function

AUTHORS: Balazs Palhazi, Alireza Paikari, Greta Clarén, Silke Appenzeller, Fabian Imdahl, Christoph Daniel, Matthias Dr Becker and Manfred Gessler

Dear Dr Becker,

I have now received all the referees reports on the above manuscript, and have reached a decision. The referees' comments are appended below.

The overall evaluation is positive and we would like to publish a revised manuscript in Development, provided that the referees' minor comments can be satisfactorily addressed. Please attend to all of the reviewer 3 comments in your revised manuscript. If you do not agree with any of their criticisms or suggestions explain clearly why this is so. If it would be helpful, you are welcome to contact us to discuss your revision in greater detail. Please send us a point-by-point response indicating your plans for addressing the referees' comments, and we will look over this and provide further guidance.

Reviewer 1

SUMMARY OF THE ADVANCE MADE IN THIS PAPER AND ITS POTENTIAL SIGNIFICANCE TO THE FIELD

The authors have addressed all issues to my satisfaction.

Reviewer 2

SUMMARY OF THE ADVANCE MADE IN THIS PAPER AND ITS POTENTIAL SIGNIFICANCE TO THE FIELD

The authors addressed all my questions and suggestions in a satisfactory manner.

© 2026. Published by The Company of Biologists under the terms of the Creative Commons Attribution License (https://creativecommons.org/licenses/by/4.0/).

SUGGESTIONS TO AUTHORS

I have no further issues that should be addressed.

Reviewer 3

SUMMARY OF THE ADVANCE MADE IN THIS PAPER AND ITS POTENTIAL SIGNIFICANCE TO THE FIELD

This manuscript demonstrates that early, nephron progenitor-specific loss of TRIM28 disrupts progenitor maintenance, accelerates differentiation programs, and leads to perinatal lethality without tumor formation in mice. By integrating single cell analyses with human Wilms tumor data, the authors provide a coherent developmental explanation for why TRIM28 loss promotes tumorigenesis in humans but not in early mouse models. Overall, the study offers a careful and conceptually sound framework that clarifies the context dependent role of TRIM28 in kidney development and disease.

SUGGESTIONS TO AUTHORS

1. Please ensure that all abbreviations are defined in each figure legend and that panels are fully self-explanatory.
2. While the conclusions regarding impaired kidney function are well supported by morphological and transcriptomic data, consider adding a brief clarifying sentence (in the Abstract or Discussion) explicitly noting that functional impairment is inferred from structural disruption and gene expression signatures rather than from direct physiological assays.
3. The manuscript presents compelling evidence for cell type specific TE deregulation following TRIM28 loss. Briefly reinforcing how these findings relate to established models of TRIM28/KRAB ZFP mediated enhancer control during differentiation—potentially with one additional key reference—could further strengthen the conceptual context without adding experimental burden.
4. Minor consistency and clarity checks
   a. Ensure that sample sizes (N) are consistently stated across all figure legends and supplementary materials.
   b. Confirm uniform terminology for nephron progenitor subpopulations and proximal tubule stages across the Results and Discussion.
   c. A final pass for minor typographical or stylistic inconsistencies would improve overall readability.
5. Some studies that would be valuable additions to the manuscript for contextualizing and strengthening its findings:
   a. Turelli P. et al. Interplay of TRIM28 and DNA methylation in controlling human endogenous retroelements. Genome Research 2014
   b. Short K. et al. Global quantification of tissue dynamics in the developing mouse kidney. Development 2014
   c. Chuong EB et al. Regulatory activities of transposable elements: from conflicts to benefits. Nature Reviews Genetics 2016

Second revision

Author response to reviewers' comments

Reviewer 3:

Potential significance to the field:

This manuscript demonstrates that early, nephron progenitor-specific loss of TRIM28 disrupts progenitor maintenance, accelerates differentiation programs, and leads to perinatal lethality without tumor formation in mice. By integrating single cell analyses with human Wilms tumor data,

© 2026. Published by The Company of Biologists under the terms of the Creative Commons Attribution License (https://creativecommons.org/licenses/by/4.0/).

the authors provide a coherent developmental explanation for why TRIM28 loss promotes tumorigenesis in humans but not in early mouse models. Overall, the study offers a careful and conceptually sound framework that clarifies the context dependent role of TRIM28 in kidney development and disease.

Response: We thank the reviewer for this positive comment on our study

Response to suggestions to authors:

1. Please ensure that all abbreviations are defined in each figure legend and that panels are fully self-explanatory.

Response: We carefully reviewed all figure legends and panels to ensure that all abbreviations are defined and that each panel is fully self-explanatory.

2. While the conclusions regarding impaired kidney function are well supported by morphological and transcriptomic data, consider adding a brief clarifying sentence (in the Abstract or Discussion) explicitly noting that functional impairment is inferred from structural disruption and gene expression signatures rather than from direct physiological assays.

Response: Thank you for raising this important point we have now included a paragraph addressing this issue in the discussion section. We now write on page 14 lines 22-27: "This defect, in conjunction with the additional defects in the tubular and glomerular systems described in this study, may account for the early lethality of Trim28$^{\Delta/\Delta}$ animals. However, as this conclusion is based solely on structural abnormalities and transcriptomic alterations rather than direct physiological measurements, future studies should incorporate in vivo functional assays to substantiate this hypothesis." (The added text is highlighted in bold magenta)

3. The manuscript presents compelling evidence for cell type specific TE deregulation following TRIM28 loss. Briefly reinforcing how these findings relate to established models of TRIM28/KRAB ZFP mediated enhancer control during differentiation—potentially with one additional key reference—could further strengthen the conceptual context without adding experimental burden.

Response: We have now included a paragraph addressing this issue in the Discussion section and now write on page 15 lines 14-22: "The cell type-specific TE deregulation reported here is consistent with the role of TRIM28 and KRAB-ZFPs in enforcing epigenetic silencing of transposable elements during differentiation (Turelli et al., 2014). In addition to the silencing of TEs, there is an increasing recognition of their role as a prolific source of cis-regulatory elements, including enhancers and transcription factor binding sites, which are co-opted for the regulation of host genes (Chuong et al., 2017). This suggests that the deregulation of TEs upon TRIM28 loss may have broader consequences for the transcriptional identity of the developing kidney.." (The added text is highlighted in bold magenta)

4. Minor consistency and clarity checks
a. Ensure that sample sizes (N) are consistently stated across all figure legends and supplementary materials.

Sample sizes are now stated consistently across all figure legends and supplementary materials.

b. Confirm uniform terminology for nephron progenitor subpopulations and proximal tubule stages across the Results and Discussion.

We reviewed the manuscript and ensured that uniform terminology is used for nephron progenitor subpopulations and proximal tubule stages throughout the Results and Discussion sections. The corresponding changes are highlighted in green.

c. A final pass for minor typographical or stylistic inconsistencies would improve overall readability.

© 2026. Published by The Company of Biologists under the terms of the Creative Commons Attribution License (https://creativecommons.org/licenses/by/4.0/).

We asked a native English speaker to perform a final review for minor typographical and stylistic inconsistencies. Changes resulting from this review are highlighted in blue in the main text.

5. Some studies that would be valuable additions to the manuscript for contextualizing and strengthening its findings:
a. Turelli P. et al. Interplay of TRIM28 and DNA methylation in controlling human endogenous retroelements. Genome Research 2014
b. Short K. et al. Global quantification of tissue dynamics in the developing mouse kidney. Development 2014
c. Chuong EB et al. Regulatory activities of transposable elements: from conflicts to benefits. Nature Reviews Genetics 2016

Response: We thank the reviewer for these valuable suggestions. We have now incorporated the references by Turelli et al. (2014) and Chuong et al. (2016) in the new Discussion paragraph on page 15, lines 14-22 (see response to Comment 3).

To incorporate the citation by Short et al. (2014), we added a new paragraph discussing the potential impact of stage-specific nephron progenitor cell (NPC) proliferation on our findings. We now state in the Discussion section on page 12, lines 8-22: "In this study, we examined the impact of Trim28 loss on the development of the mouse kidney. We found that homozygous loss of Trim28 in the nephrogenic lineage resulted in postnatal lethality and a reduced kidney-to-body weight ratio. This reduction in kidney mass may be due to the smaller NPC population that we observed at developmental stages E17.5 and E18.5. The smaller progenitor pool may become exhausted more rapidly and consequently generates fewer nephron structures. However, in contrast to the reduced NPC population size, we observed that the fraction of proliferating NPCs was comparable between Trim28$^{\Delta/\Delta}$ and Trim28f/f animals at these later developmental stages. A previous report (Short et al., 2014) demonstrated that NPC proliferation is strongly stage-dependent, with higher proliferation rates at earlier developmental stages (e.g., E13.25) than at later stages (e.g., E17.25). Therefore, it is possible that NPC proliferation is impaired at earlier developmental time points in Trim28$^{\Delta/\Delta}$ animals compared to Trim28f/f controls. Future studies should therefore include earlier developmental stages in order to fully assess the impact of Trim28 loss on NPC proliferation dynamics."

We sincerely thank the reviewer for their constructive criticism and helpful suggestions, which have significantly improved the manuscript.

Third decision letter

MS ID#: dev.205188R2

MS TITLE: The absence of Trim28 in nephron progenitors results in impaired kidney development and function

AUTHORS: Balazs Palhazi, Alireza Paikari, Greta Clarén, Silke Appenzeller, Fabian Imdahl, Christoph Daniel, Matthias Dr Becker and Manfred Gessler

Dear Dr Becker,

I am happy to tell you that your manuscript has been accepted for publication in Development, pending our standard publication integrity checks.

© 2026. Published by The Company of Biologists under the terms of the Creative Commons Attribution License (https://creativecommons.org/licenses/by/4.0/).
